# Age-associated G-quadruplex accumulation and DDX5 loss shape chromatin landscapes in human astrocytes

**DOI:** 10.21203/rs.3.rs-7933297/v1

**Published:** 2025-11-19

**Authors:** Andrey Tsvetkov, Vijay Kumar M J, Rocio Diaz Escarcega, Ellery Wheeler, Nitin Tandon, David Monchaud, Christopher Hartl

**Affiliations:** The University of Texas McGovern Medical School at Houston; The University of Texas McGovern Medical School at Houston; The University of Texas McGovern Medical School at Houston; The University of Texas McGovern Medical School at Houston; The University of Texas McGovern Medical School at Houston; UBE DIjon; The Epigenome Technologies

**Keywords:** G-quadruplex, DDX5, G4 CUT&Tag, ATAC-seq, astrocytes, chromatin remodeling, G4 homeostasis, aging

## Abstract

Aging disrupts genome organization and transcriptional fidelity, but the role of non-canonical DNA structures in the aging process remains unclear. G-quadruplexes (G4s), stable guanine-rich DNA and RNA structures are established regulators of gene expression and genome integrity, yet their contribution to physiological aging is unknown. Using fluorescent imaging with primary human astrocytes derived from individuals spanning early to late adulthood (22–73 years) reveals an accumulation of G4s and a reduced nuclear expression of the G4-resolving helicase DDX5 in aging cells. To investigate how these changes relate to genome architecture, we performed ATAC-seq to profile chromatin accessibility and G4 CUT&Tag to profile the G4 landscape across all astrocyte cultures. Older cells exhibited global chromatin compaction and focal G4 enrichment, with gains occurring in both accessible and closed chromatin regions, indicating *locus*-specific and context-dependent regulation. To determine whether DDX5 modulates these features, we overexpressed DDX5 in young astrocytes and identified transcriptional targets involved in chromatin organization and genome maintenance. Acute DDX5 knockout caused focal G4 accumulation without widespread chromatin changes, indicating that DDX5 maintains and modulates G4 dynamics at defined genomic regions. Together, these findings reveal G4s as dynamic, age-sensitive features of the genome with potential roles in epigenetic regulation and establish DDX5 as a modulator of G4 dynamics and genome integrity during human brain aging.

## Introduction

Aging is a complex biological process marked by progressive changes in genome regulation, chromatin organization, and gene expression^1–7^. These alterations affect core cellular functions such as transcription, DNA repair, RNA processing, and epigenetic remodeling, gradually compromising cellular integrity^2, 3, 8–11^. Epigenetic marks, including DNA methylation and histone modifications, undergo gradual shifts that influence gene expression^10, 12–14^. Aging is also accompanied by extensive chromatin remodeling, disturbing long-range chromatin interactions, chromatin loops, and topologically associated domains, thereby impairing coordinated gene regulation and genome integrity, contributing to phenotypic heterogeneity among cells^8, 15–20^. In parallel, chromatin accessibility becomes uneven, with certain genomic regions becoming more relaxed and accessible while others exhibit increased compaction, reflecting a *locus*-specific remodeling pattern that alters regulatory potential across the genome. These multi-layered genome changes accumulate gradually, reshaping the chromatin and transcriptional landscape in a way that predisposes cells and tissues to dysfunction over time^1, 21–25^. While much of aging research has focused on how these processes are affected at the level of DNA and RNA, the contribution of higher-order nucleic acid structures remains unclear.

G-quadruplexes are four-stranded nucleic acid structures formed in guanine (G)-rich regions of DNA and RNA^26–33^. G4s are structurally stable and regulate key cellular processes such as replication^34^, transcription^35, 36^, translation^37^, telomere maintenance, and genome stability^28, 38–41^. G4s are widespread throughout the genome (covering *ca*. 1% of autosomes^42^), with high density near gene promoters, untranslated regions, replication origins, enhancers, and telomeres, suggesting a regulatory role at sites critical for transcriptional control and gene expression^29, 43–55^. The dynamics of G4s are modulated and regulated by specific proteins. G4 helicases resolve G4s, to enable smooth replication and transcriptional elongation, for instance, and G4-binding proteins mold G4s, to promote site-specific regulatory functions^30, 56–64^. Dysregulation of G4 homeostatic balance, caused either by overly stabilized G4s or compromised function of G4 helicases, can interfere with polymerase progression, disrupt transcriptional fidelity, and contribute to genomic instability^35, 58, 65–68^. While G4s have been widely studied in yeast cells^69^ and cancer^70–75^, their function and significance in the context of aging remain poorly understood^26^. In particular, it is not known how G4 dynamics and their regulation shift with age and how they influence transcriptional and broader epigenetic changes over time. Identifying the molecular mechanisms that control G4 homeostasis during aging may provide new insight into how genome regulation is altered over the lifespan.

G4 helicases and G4-binding proteins have been directly linked to several genome maintenance and premature aging disorders^26, 49, 76, 77^. Several helicases, including Werner syndrome helicase (WRN), Bloom syndrome protein (BLM), DEAD-box helicase 5 (DDX5), Fanconi Anemia group J protein (FANCJ), Regulation of Telomere Elongation Helicase 1 (RTEL1), DEAH-box helicase 36 (DHX36), and RecQ-like helicase 4 (RECQL4) actively unwind G4s to prevent replication stress, transcriptional interference and genome instability^78^. Mutations in these helicases are associated with Werner syndrome (WRN)^79–82^, Bloom syndrome (BLM)^83–88^, Fanconi anemia (FANCJ)^89–93^, Hoyeraal–Hreidarsson syndrome (RTEL1)^94–98^, and Rothmund–Thomson syndrome (RECQL4)^99–101^. These syndromes share features of genomic instability, senescent phenotypes, and accelerated aging, highlighting the essential role of G4 regulation in maintaining genome integrity. While the roles of G4 helicases in development and cancer are well established, their specific functions and regulatory mechanisms in physiological aging remain poorly understood. Elucidating how these proteins control G4 homeostasis may reveal new mechanisms by which chromatin structure and transcriptional balance are maintained across the human lifespan.

To investigate how G4 structures are regulated during aging, we used a unique model of eight primary human astrocyte cultures derived from brain tissue isolated during epilepsy surgeries, representing individual samples aged 22, 24, 32, 34, 53, 56, 72, and 73 years. Across this spectrum, astrocytes displayed an age-associated senescence profile with elevated p16, p21, and p53 levels, a progressive increase in G4 signal detected by BG4, a G4-specific antibody^102, 103^, and N-TASQ, a fluorescent G4-ligand^104^, in a pattern consistent with a G4 clock and reduced nuclear expression of the G4-helicase DDX5, a major G4-resolving RNA helicase^105–111^. ATAC-seq (for Assay for Transposase Accessible Chromatin) revealed a progressive, age-associated chromatin compaction throughout the examined age range, with accessibility consistently reduced from 22 to 73 years. In contrast, G4 CUT&Tag (for Cleavage Under Targets and Tagmentation) showed variable focal G4 enrichment. G4 occupancy was highest in individuals aged 72 and 73 years, and younger individuals showed considerable variability. Integrative regression analysis identified multiple distinct categories of age-sensitive *loci*, some showing both G4 accumulation and chromatin closure, while others displayed G4 gain with increased accessibility, suggesting a context-dependent relationship between G4s and chromatin state. Given the age-associated increase in G4 occupancy and the concurrent downregulation of DDX5, we investigated whether DDX5 regulates G4 dynamics and genome function. We overexpressed DDX5 in young astrocytes and performed RNA-seq, revealing differential expression of genes involved in chromatin remodeling, DNA topology, and cell cycle regulation. To directly assess DDX5’s role in chromatin structure and G4 regulation, we generated DDX5 knockdown astrocytes and performed ATAC-seq and G4 CUT&Tag, which showed focal G4 accumulation with minimal changes in global chromatin accessibility. Together, these findings identify G4 structures as dynamic, age-responsive genomic features and establish DDX5 as an important regulator of G4 homeostasis and transcription during human brain aging.

## Results

### Human astrocytes as an in vitro model system of cellular aging

**Figure 1:**
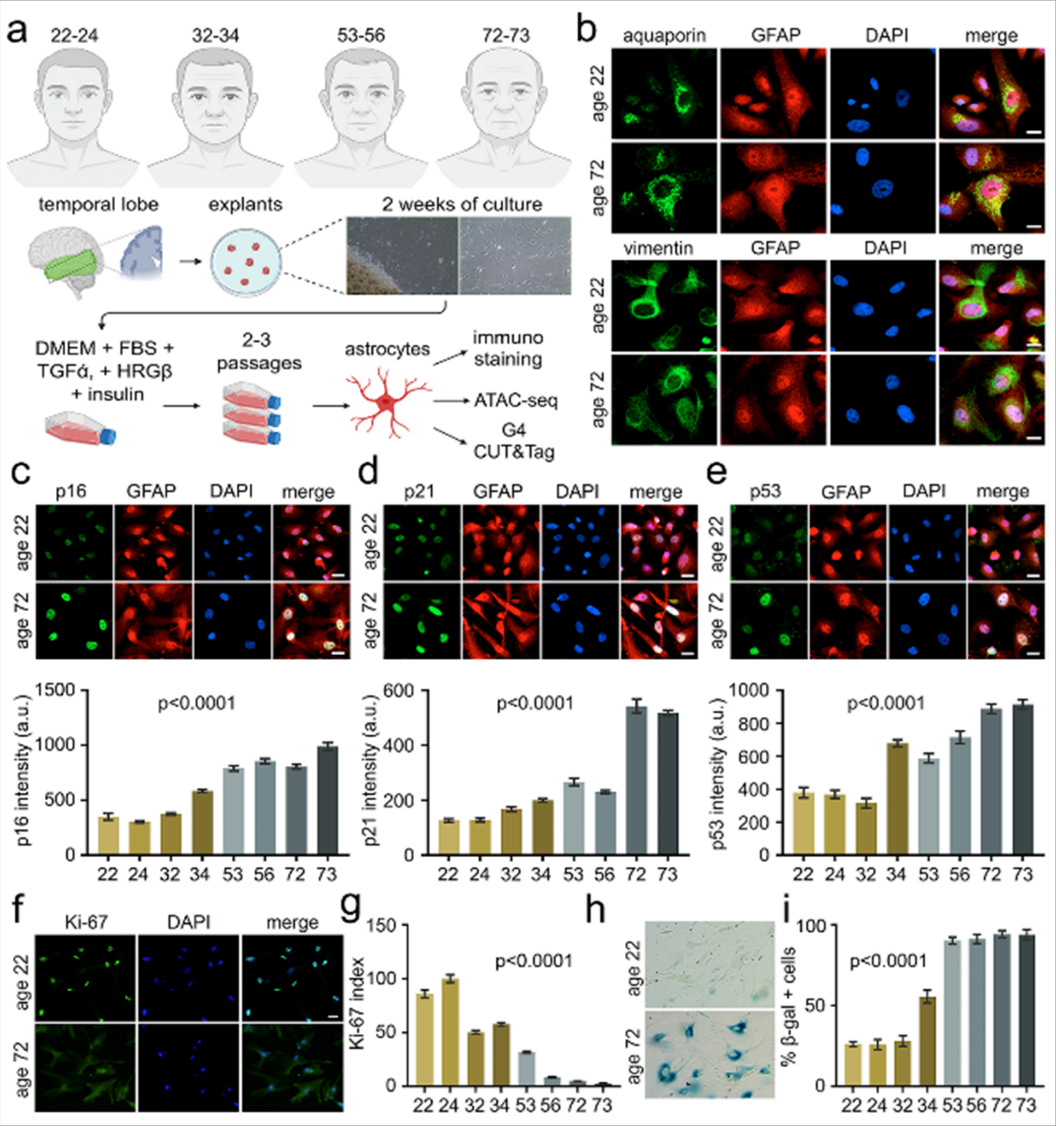
Human astrocytes as an *in vitro* model system of cellular aging. **(a)** A schematic overview of the workflow used to isolate and culture human astrocytes from the frontal lobe epilepsy surgical resections of individuals from age groups 22–24, 32–34, 53–56, and 72–73 years. **(b)** The purity of human astrocyte cultures was checked by staining for astrocyte-specific markers, aquaporin and vimentin (green), GFAP (red), and nuclear dye DAPI (blue). N = 8 (ages: 22–male, 24–male, 32–male, and 34–male, 53–male, 56–female, 72–male, and 73–female). Samples were imaged with a confocal microscope. Images from age 22 and age 72 are used for representation. Scale bar, 10 μm. All the cells were positive for astrocyte markers, indicating the purity of the cultures. **(c)** Human astrocytes from age groups 22–24, 32–34, 53–56, and 72–73 years were stained for senescence markers p16 (green), GFAP (red), and nuclear DAPI (blue). The compiled graph below displays increased p16 across the age groups. **(d)** Astrocytes from age groups 22–24, 32–34, 53–56, and 72–73 years were stained for p21 (green), GFAP (red), and nuclear DAPI (blue). The graph below shows increased levels of p21 with age. **(e)** Astrocytes were stained for p53 (green), GFAP (red), and DAPI (blue) from age groups 22–24, 32–34, 53–56, and 72–73 years. The graph below displays levels of p53 across age groups. Images from age 22 and age 72 are used for representation. Samples were imaged with a confocal microscope, and intensities were measured and normalized with ImageJ. Scale bar, 10 μm. Data are presented as mean ± SEM. (****p < 0.0001, unpaired two-tailed *t-test*). >750 astrocytes per age group were analyzed. **(f)** ICC for the proliferation marker Ki-67 in astrocytes from age groups 22–24, 32–34, 53–56, and 72–73 years. A marked reduction in Ki-67–positive cells was observed in cultures from the age group 53–73. Scale bar, 20 μm. **(g)** Quantification of the Ki-67 index in the cells in all 8 age groups. Data are shown as mean ± SEM. (****p < 0.0001, unpaired two-tailed t-test). **(h)** Representative brightfield images of senescence-associated β-galactosidase (SA-β-gal) staining in astrocytes from age groups 22–24, 32–34, 53–56, and 72–73 years. **(h)** Percentage of SA-β-gal–positive cells in cells across age groups. Data are shown as mean ± SEM. (****p < 0.0001, unpaired two-tailed t-test).

Astrocytes were cultured from explants derived from the temporal lobe of human brain tissue resections obtained from epilepsy patients. Explants were generated from individuals aged 22–male, 24–male, 32–male, 34–male, 53–male, 56–female, 72-male, 73-female, and were processed for astrocyte culture *in vitro* in astrocyte-specific media supplemented with growth factors (Fig. 1a). The astrocytic phenotype of these isolated cells was rigorously confirmed through immunostaining, utilizing a panel of well-established astrocyte-specific markers, including glial fibrillary acidic protein (GFAP), aquaporin, vimentin (Fig. 1b), excitatory amino acid transporter 2 (EAAT2), and S100β **(Figure S1a**–**1b)**. Human astrocytes were positive for all the astrocyte-specific markers, confirming the purity of the cultures. Astrocyte cultures from all age groups were used at early passages (3–4) to avoid culture-induced senescence.

To evaluate the senescence profiles of human astrocytes, we immunostained astrocytes of all eight age groups with established senescence markers, p16, p21, along with p53, a key upstream regulator of the senescence pathway^112^. Our results displayed significantly elevated expression levels of p16, p21, and p53 in age groups of 53, 56, 72, and 73 compared to the age groups of 22, 24, 32, and 34 (Fig. 1c–1h). To further score for DNA damage, we conducted immunostaining of astrocytes with γH2AX **(Figure S1c, 1e)** and 53BP1 **(Figure S1d–S1f)**. Our observations highlighted that astrocytes from age groups of 53, 56, 72, and 73 exhibited marked increases in γH2AX and 53BP1 levels, indicating that a senescent state of astrocytes is associated with DNA damage.

To assess cellular proliferation across age groups, we immunostained human astrocytes with Ki-67, a well-established marker of proliferation^113^. Astrocytes derived from individuals aged 53, 56, 72, and 73 exhibited a marked reduction in the proportion of Ki-67–positive nuclei compared to astrocytes from ages 22, 24, 32, and 34, indicating a decline in proliferative capacity with increasing age (Fig. 1i). We next evaluated lysosomal β-galactosidase activity using senescence-associated β-galactosidase (SA-β-gal) staining. Astrocytes from ages 53, 56, 72, and 73 showed a robust increase in SA-β-gal–positive cells, whereas astrocytes from ages 22, 24, 32, and 34 displayed minimal SA-β-gal staining (Fig. 1j). These findings show that astrocytes demonstrate age-associated changes in proliferation and replicative senescence activity. We thus developed an *in vitro* model where we observed older age groups (53, 56, 72, and 73) exhibiting senescence phenotypes, and thus provide a novel and unique platform to investigate age-associated changes in G4 homeostasis, chromatin architecture, and transcriptional regulation, offering novel insights into the epigenetic mechanisms underlying human brain aging.

#### Age-associated accumulation of G4 structures and reduced DDX5 expression in human astrocytes

**Figure 2:**
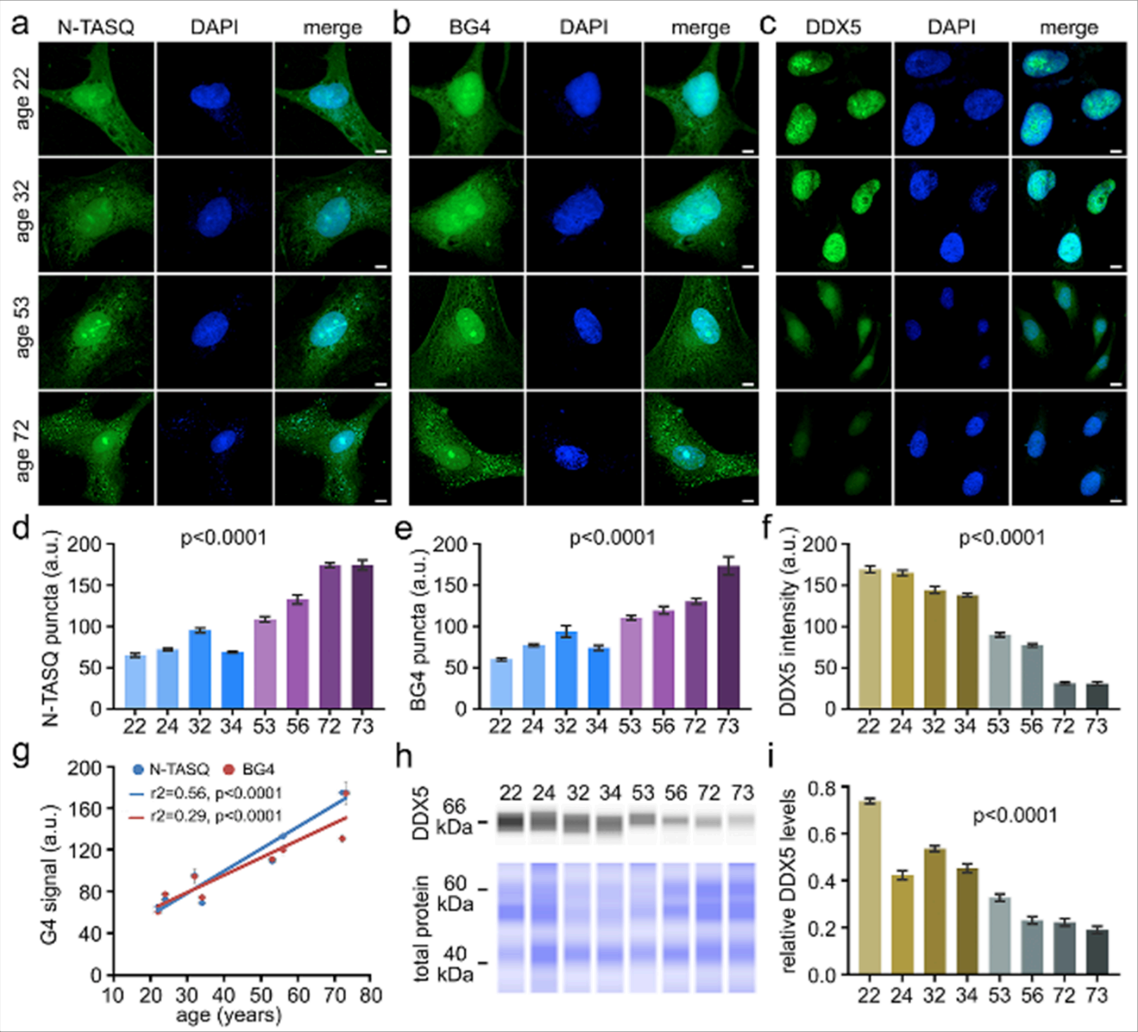
Age-associated accumulation of G4 structures and reduced DDX5 expression in human astrocytes. **(a)** Primary human astrocytes from individuals aged 22–24, 32–34, 53–56, and 72–73 years were fixed and stained with 25 μM N-TASQ (green) to detect G4 structures, along with the nuclear dye DAPI (blue). N-TASQ–positive G4 puncta were predominantly nuclear with small cytoplasmic punctate (G4-RNA) clusters enriched in astrocytes from the 53–73-year age group. Samples were imaged with a confocal microscope. Scale bar, 5 μm. Representative images from ages 22, 32, 53, and 72 are shown. **(b)** ICC for G4 structures using the BG4 antibody (green), with DAPI (blue), in astrocytes from the same eight individual samples. BG4-positive G4 puncta were observed in the nucleus, with cytoplasmic puncta (G4-RNA) in astrocytes from samples aged 53–73. Samples were imaged with a confocal microscope. Scale bar, 5 μm. Representative images from ages 22, 32, 53, and 72 are shown. **(c)** ICC for DDX5 (green) with DAPI (blue) in astrocytes from all eight individual samples. DDX5 is primarily nuclear, and its expression is reduced in cells from the 53–73-year age group. Samples were imaged with a confocal microscope. Scale bar, 10 μm. Representative images from ages 22, 32, 53, and 72 are shown. **(d)**Quantification of N-TASQ nuclear puncta intensity in astrocytes from all eight individual samples (ages 22, 24, 32, 34, 53, 56, 72, and 73). Intensity values were measured using ImageJ and normalized across samples. A significant increase in the G4 signal was observed in cells across age groups from 53–73 years. >250 astrocytes per group were analyzed. Data represent mean ± SEM. ****p < 0.0001 (one-way ANOVA with Tukey’s *post-hoc* test). **(e)**Quantification of BG4 nuclear puncta intensity in astrocytes from all eight individuals, measured and normalized using ImageJ. There is a significant increase in the G4 signal with age. >250 cells per group analyzed. Data represent mean ± SEM. ****p < 0.0001 (one-way ANOVA with Tukey’s *post-hoc* test). **(f)**Quantification of DDX5 nuclear intensity across all eight age groups. There is a significant age-dependent decrease in the expression of DDX5 in human astrocytes. >750 astrocytes per group were analyzed. Data represent mean ± SEM. ****p < 0.0001 (one-way ANOVA with Tukey’s *post-hoc* test). **(g)**Linear regression analysis of G4 signal intensity (from N-TASQ and BG4 staining) across the eight age groups. Both N-TASQ and BG4 staining show a significant positive correlation between individual age and G4 accumulation (N-TASQ: r^2^ = 0.56, p < 0.0001; BG4: r^2^ = 0.29, p < 0.0001). **(h)**DDX5 protein levels were measured across all eight astrocyte samples. by capillary-based immunoassay (ProteinSimple, Bio-techne). Total protein stain was used for normalization. **(i)** Quantification of DDX5 protein levels across age groups. DDX5 shows a significant age-dependent reduction in human astrocytes across age groups. Data represent mean ± SEM. **p < 0.01 (unpaired two-tailed *t-test*).

To investigate age-associated changes in the G4s state in human astrocytes, we employed two methodologies. First, we used NaphthoTASQ (N-TASQ), a highly specific G4 ligand and fluorescent probe capable of detecting both DNA and RNA G4s^30, 44, 104, 114, 115^. Fixed human astrocytes from all eight age groups were stained with N-TASQ, revealing that cells from individuals aged 53, 56, 72, and 73 years contained more stable G4 structures, as evidenced by increased G4-positive puncta in both the nucleus and cytoplasm (Fig. 2a, 2d). Second, we employed the BG4 antibody, which selectively recognizes G4 structures in fixed cytological specimens^102^. BG4 staining also revealed a substantial increase in the G4 structures in astrocytes from ages 53, 56, 72, and 73 compared to astrocytes from ages 22, 24, 32, and 34 (Fig. 2b, 2e). Both N-TASQ and BG4 revealed large nuclear *foci* and distinct cytoplasmic clusters, highlighting the presence of G4-enriched structures in astrocytes across age groups. To quantitatively evaluate the relationship between G4 abundance and age, we performed regression analysis using N-TASQ and BG4 staining data (Fig. 2g). This analysis demonstrated a strong positive correlation between age and G4 accumulation, indicating that more stable G4 structures increase with age.

G4 abundance is finely regulated in cells by helicases. One of them, DDX5, which maintains transcription^105–107, 109, 116–118^, genome stability^108^, chromatin remodeling^110^, and RNA processing^109, 119^, has also been implicated in aging. Recent work showed that loss of DDX5 in cartilage promotes fibrosis through failure to unwind G4s^120^, while another study demonstrated that DDX5 forms prion-like condensates in the brain that accumulate with age, indicating altered helicase function during aging^121^. However, whether DDX5 dysfunction contributes to aberrant G4 accumulation and impaired chromatin regulation in the aging process remains unknown, highlighting an unaddressed link between helicase activity, G4 homeostasis, and age-associated chromatin remodeling. To address this, we measured DDX5 expression in human astrocytes and observed a strong nuclear staining in samples from ages 22, 24, 32, and 34, with markedly reduced expression in astrocytes from ages 53, 56, 72, and 73 (Fig. 2c, 2f). The DDX5 expression was further confirmed by JESS-based capillary immunoassay, which showed lower protein levels in astrocytes from ages 53, 56, 72, and 73 (Fig. 2h, 2i). Together, these findings indicate an age-related reduction in DDX5 helicase function, which may contribute to the accumulation of G4 structures during aging.

#### Chromatin accessibility decreases with age in human astrocytes

**Figure 3:**
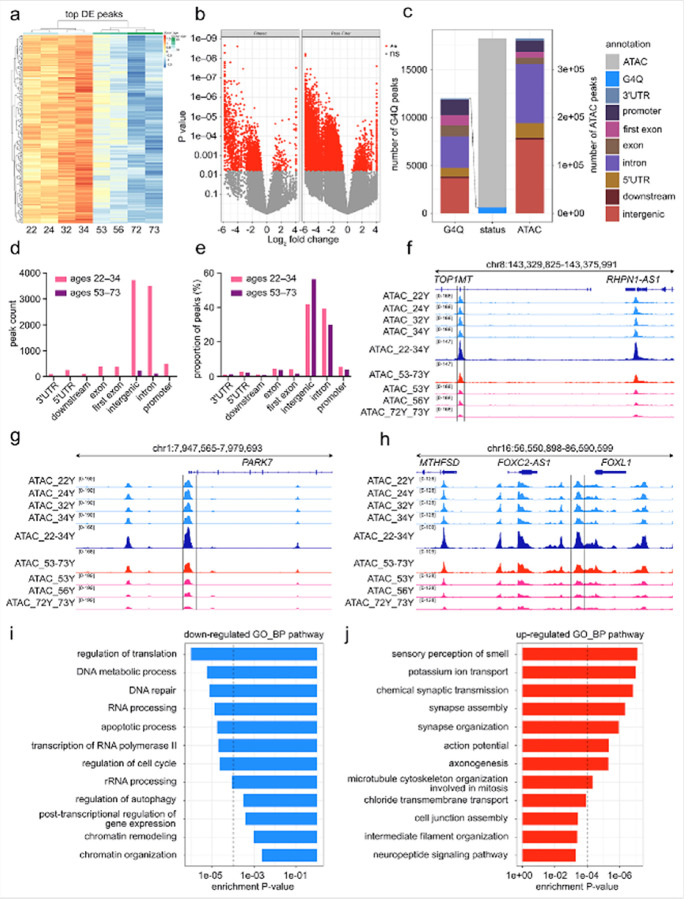
Chromatin accessibility decreases with age in human astrocytes. **(a)** Heatmap showing hierarchical clustering of the top differentially accessible chromatin regions identified by ATAC-seq (FDR ≤ 10%) in astrocytes from eight individuals aged 22, 24, 32, 34, 53, 56, 72, and 73 years. Accessibility values were z-score normalized across samples. **(b)** Volcano plots showing differential chromatin accessibility across all ATAC-seq peaks between astrocytes from samples aged 22, 24, 32, and 34 versus 53, 56, 72, and 73 years. Each dot represents an individual peak, plotted as log_2_ fold change versus −log_10_(p-value). Peaks with FDR ≤ 10% are highlighted in red. Left: filtered dataset; right: post-filtering and normalization. **(c)** Stacked bar plot showing the distribution of genomic **annotations** for all called peaks from ATAC-seq and G4 CUT&Tag datasets. Peaks were assigned to categories such as intergenic, intron, promoter, UTR, and exon. The G4Q and ATAC bars display the relative number of peaks in each annotation group. The central status bar reflects the total peak counts for each assay. Y-axes are scaled independently: the left y-axis corresponds to G4 peaks (up to ~15,000), and the right y-axis corresponds to ATAC peaks (up to ~300,000). **(d)** Bar plot showing the number of differentially accessible peaks assigned to each annotation category, including promoter, intron, intergenic, exon, UTRs, and downstream regions. The majority of peaks with higher accessibility in the 22, 24, 32, and 34 years were mapped to intergenic and intronic regions, whereas relatively fewer peaks were enriched in these regions in the 53, 56, 72, and 73 years. **(e)** Bar plot showing the % of proportion of differentially accessible peaks assigned to each genomic category for 22, 24, 32, and 34 and 53, 56, 72, and 73 years. **(f)** Genome browser view (IGV) showing ATAC-seq signal tracks at the ***TOP1MT–RHPN1-AS1*** locus in human astrocytes from all eight age groups. A clear reduction in chromatin accessibility is observed across this region in the samples from 53, 56, 72, and 73 years compared to the 22, 24, 32, and 34 years, consistent with age-associated loss of accessibility at this *locus*. **(g)** IGV plot of the ***PARK7*** *locus* in human astrocytes from eight age groups. **(h)** IGV plot for ***MTHFSD–FOXC2-AS1–FOXL1*** region. Accessibility peaks in the samples from 22, 24, 32, and 34 years are sharply reduced compared to samples from 53, 56, 72, and 73 years, indicating a focal, age-associated decline in chromatin accessibility at this locus. Samples from ages 22, 24, 32, and 34 are shown in blue, and those from 53, 56, 72, and 73 in red. **(i)** Bar plot showing Gene Ontology (GO), Biological processes (BP) enrichment analysis of genes associated with chromatin regions with reduced accessibility in astrocytes. Enrichment p-values are shown on the x-axis. **(j)** GO: BP enrichment analysis of genes associated with chromatin regions that show increased accessibility. Enrichment p-values are shown on the x-axis.

G4 formation is also known to be dependent on chromatin status^36, 58, 122–125^. To investigate this, we performed ATAC-seq on human astrocytes from eight individuals (ages 22, 24, 32, 34, 56, 56, 72, and 73) to assess age-associated changes in chromatin accessibility. To identify genome-wide patterns linked to age, we selected the top differentially accessible peaks (false discovery rate (FDR) ≤ 10%) and performed hierarchical clustering. Surprisingly, the resulting heatmap revealed a clear age-dependent segregation, with astrocytes from ages 22, 24, 32, and 34 years clustering distinctly from those aged 53, 56, 72, and 73 years (Fig. 3a, S2b). Notably, several genomic regions that showed strong accessibility in astrocytes from the 22, 24, 32, and 34 ages exhibited a progressive decline in signal in astrocytes from the 53, 56, 72, and 73 ages, indicating a gradual age-associated loss in chromatin accessibility at shared genomic *loci*. We thus performed a differential analysis comparing astrocytes from samples aged 53, 56, 72, and 73 to those from 22, 24, 32, and 34 years. A large number of genomic regions showed significantly reduced chromatin accessibility in astrocytes from the 53, 56, 72, and 73 ages, whereas only a smaller set of regions became more accessible with age. (Fig. 3b). To test whether these changes occurred progressively with age, we performed a regression analysis treating individual age as a continuous variable. Most significant peaks showed a negative age-coefficient consistent with a progressive decline in accessibility with increasing age **(Figure S3a)**.

The chromatin accessibility near the transcription start sites (TSS) was progressively reduced in astrocytes throughout the age groups. Astrocytes from individuals aged 22, 24, 32, and 34 showed stronger and broader signal distribution within the ± 2 kb window, whereas cells from individuals aged 53, 56, 72, and 73 years exhibited a marked reduction in promoter-proximal accessibility **(Figure S2a)**. These changes mirror the patterns observed at distal regulatory elements and support a global decline in chromatin accessibility with age. To visualize global differences in chromatin accessibility, we examined the distribution of normalized ATAC-seq counts using violin plots. Astrocytes from samples aged 22, 24, 32, and 34 showed overall higher chromatin accessibility than astrocytes from individuals aged 53, 56, 72, and 73 **(Figure S2c)**. This pattern indicates that chromatin accessibility decreases progressively with age and that the reduction affects a broad range of genomic regions.

Next, we annotated ATAC-seq peaks to evaluate their genomic distribution and how they change with age. Most peaks were located in intergenic and intronic regions, followed by promoters and first exons (Fig. 3C). Age-enriched peaks in astrocytes from ages 22, 24, 32, and 34 were predominantly intergenic and intronic, while very few peaks were enriched in samples from ages 53, 56, 72, and 73 years among any annotation category (Fig. 3D). When adjusted for total peak number, intergenic peaks accounted for a larger proportion in the samples from 53, 56, 72, and 73 years, whereas intronic peaks were more common in the 22-, 24-, 32-, and 34-years old samples (Fig. 3E). These results indicate that age-related chromatin accessibility loss occurs in diverse genomic regions, with intergenic elements relatively preserved in later ages. We used an UpSet plot to examine how accessible chromatin regions were shared or unique among astrocytes from individual samples of different ages **(Figure S2d).** While there were some peaks unique to individual samples (*e.g*., the 32-year-old individual) or to only a few age groups, these were comparatively fewer in number. Overall, the plot shows that age-associated differences in accessibility are largely reflected in variations in the shared chromatin landscape, with only a small fraction of sites being unique to specific ages **(Figure S2d)**.

Taken together, these results demonstrate a progressive, *locus*-specific reduction in chromatin accessibility during aging in human astrocytes. The changes occur at both promoter-proximal and distal regulatory elements, span multiple genomic compartments, and are reflected in global accessibility patterns as well as in the distribution of shared and unique peaks among individuals. Overall, the findings point to a coordinated remodeling of the chromatin landscape with age.

### Reduced chromatin accessibility at specific genomic loci reflects functional pathway changes with age in human astrocytes

To extend the genome-wide analyses, we examined ATAC-seq signal at individual *loci* throughout all individual ages to identify specific genomic regions showing marked age-associated changes in chromatin accessibility. Several genomic *loci* displayed substantial reductions in accessibility in astrocytes from ages 53, 56, 72, and 73 compared with those from ages 22, 24, 32, and 34. For example, accessibility at the *TOP1MT locus*, encoding a protein associated with mitochondrial DNA maintenance^126^, was markedly reduced in astrocytes from ages 53, 56, 72, and 73 (Fig. 3f). The *PARK7 locus*, encoding a region involved in oxidative stress^127^, also showed high accessibility in the sample individuals from 22-, 24-, 32-, and 34-year-old astrocytes but exhibited a steep decline in the 53-, 56-, 72-, and 73-year-old individuals (Fig. 3g). Similar reductions were observed at the *FOXL1 locus*, which contains regulatory elements for transcription factors^128^ (Fig. 3h), and at the *ATXN3 locus* **(Figure S3b)**, previously linked to neurodegenerative disease pathways^129^. At the *FANCI locus*, which encodes a key component of the Fanconi anemia DNA repair pathway essential for maintaining genomic stability^130^, the ATAC-seq signal was strong in astrocytes from ages 22, 24, 32, and 34 but markedly reduced in those from ages 53, 56, 72, and 73 **(Figure S3c)**. Similarly, at the *WDR70 locus*, which encodes a WD-repeat-containing protein implicated in chromatin remodeling and the DNA damage response^131^, accessibility decreases with age **(Figure S3d)**. These examples illustrate that the age-related decline in chromatin accessibility affects specific regulatory regions, including genes implicated in mitochondrial function, oxidative stress responses, genomic stability, and transcriptional regulation.

To assess the functional relevance of these chromatin accessibility changes, we performed Gene Ontology (GO) and KEGG pathway enrichment analyses on differentially accessible regions. Regions with reduced chromatin accessibility were enriched for biological processes involved, for instance, in DNA metabolic processes, DNA repair, chromatin organization, and regulation of autophagy (Fig. 3i). At the molecular function level, the regions with reduced accessibility were associated with the binding, for instance, to mRNA, transcription factors, ribosome, and histone **(Figure S3e)**. KEGG pathway analysis highlighted enrichment for genome maintenance-related pathways and longevity-regulating pathways **(Figure S3g)**. These patterns suggest that chromatin closing with age preferentially affects *loci* central to genomic stability, transcriptional control, and core cellular maintenance. Regions showing greater chromatin accessibility with age were enriched for biological processes related to chemical synaptic transmission and synapse organization (Fig. 3j). Corresponding molecular functions include the control of the activity of ion channels and G-protein coupled receptors (GPCRs) **(Figure S3f)**. KEGG pathways included neuroactive ligand-receptor interaction and neurotransmission-related signaling **(Figure S3h)**. Overall, the gene enrichment analysis indicates that chromatin accessibility changes with age in human astrocytes involve genes linked to essential cellular maintenance, genome stability, transcriptional regulation, and distinct sensory and signaling-related functions.

#### Age-associated remodeling of G4 structures in human astrocytes

**Figure 4:**
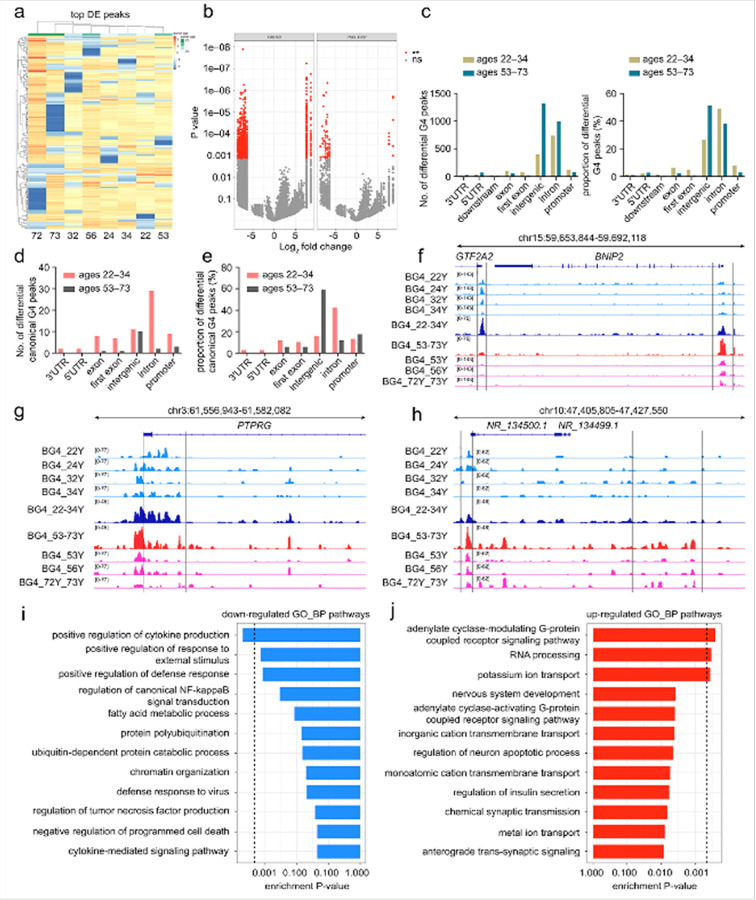
Age-associated remodeling of G4 structures in human astrocytes. **(a)** Heatmap showing z-score normalized G4 CUT&Tag signal for the top differentially enriched peaks (FDR ≤ 10%) across astrocytes from eight individuals aged 22, 24, 32, 34, 53, 56, 72, and 73 years. Each column represents an individual sample, and rows represent G4-enriched loci. Hierarchical clustering was used to organize both rows and columns based on signal similarity, highlighting age-related patterns in G4 occupancy. Individual sample age is shown using a gradient color bar. **(b)** The volcano plots showing differential peak analysis of G4 CUT&Tag data comparing astrocytes from ages 22, 24, 32, 34, and 53, 56, 72, and 73 years. Each dot represents a G4-enriched region, plotted by log_2_ fold change and p-value. Peaks that meet the FDR ≤ 10% threshold are highlighted in red. The left panel shows all detected peaks prior to filtering; the right panel shows peaks that remained after applying count-based filtering and normalization. **(c)** Genomic annotation of G4 CUT&Tag peaks that were differentially enriched between astrocyte samples from individuals aged 22, 24, 32, and 34 years and 53, 56, 72, and 73 years. Peaks were annotated based on their genomic location relative to gene features, including promoter, intron, intergenic, exon, UTRs, and downstream regions. The left panel shows the total number of differential G4 peaks assigned to each category for both age groups. The right panel displays the same data normalized as a percentage of all differential peaks per group. **(d)** Genomic distribution of differentially enriched canonical G4 peaks mapped to each genomic feature in astrocyte samples from ages 22, 24, 32, 34 years, and 53, 56, 72, 73 years. Canonical G4 peaks were defined by the presence of predicted G4 motifs overlapping with differential G4 CUT&Tag peaks. Bars represent counts of peaks assigned to promoter, intron, intergenic, exon, UTR, and first exon regions for each age group. **(e)** Percentage of canonical G4 peaks assigned to each genomic feature for all the age groups. The bar plot shows the relative distribution of peaks across categories such as promoter, intron, intergenic regions, exons, and UTRs within each group. **(f)** IGV plot for G4 signal at the ***GTF2A2–BNIP2*** *locus* in astrocytes from eight age groups: 22, 24, 32, 34, 53, 56, 72, and 73 years. Blue tracks represent samples from the 22, 24, 32, and 34 years, and red/pink tracks represent the 53, 56, 72, and 73 years. Each track shows normalized G4 signal intensity at this *locus*. **(g)** IGV track for G4 signal at the ***PTPRG*** *locus*, where G4 motifs are enriched with age. **(h)** IGV track for G4 peaks at the ***NR_134500.1–NR_134499.1***region. Tracks show normalized G4 signal intensity for each individual sample and age group averages. **(i)** Bar plot showing GO Biological Process enrichment analysis of genes associated with regions showing reduced G4 signal in astrocytes from the ages 53, 56, 72, and 73 years **(j)** GO Biological Process terms enriched among genes linked to regions with increased G4 occupancy in the 53, 56, 72, and 73 years. Enrichment p-values are plotted on the x-axis.

To complement ATAC-seq analysis, we profiled G4 structures in human astrocytes from the same eight individuals (22–73 years) using G4 CUT&Tag to examine how their genomic distribution changes with age. Hierarchical clustering of the top differentially enriched peaks (FDR ≤ 10%) revealed marked heterogeneity between individuals (Fig. 4a, S4b). The 72- and 73–year profiles showed the highest overall G4 occupancy, with strong enrichment at multiple genomic regions, although these regions were not uniformly shared between them. The 32–year profile also contained several highly enriched G4 *loci*, some overlapping with those in 72- and 73–year samples, but many were unique to the 32–year individual. The 56–year profile exhibited fewer strongly enriched *loci*, while the 24- and 34–year profiles showed a broader distribution of moderately enriched G4 peaks. The 22–year profile contained relatively few *loci* with pronounced G4 enrichment, and the 53–year profile showed fewer strongly enriched loci than the 32-, 72-, or 73–year profiles (Fig. 4a). These observations indicate that G4s accumulate most prominently in the oldest individuals at 72 and 73 years, while G4 enrichment at other ages varies substantially with distinct *loci* enriched at different points in the age range rather than a uniform shift across all individuals.

To capture systemic differences despite heterogeneity in G4 distribution, we compared the genomic distribution of G4 peaks in all individual ages to visualize both shared and age-specific G4 enrichment patterns. The analysis revealed numerous *loci* with significant variation in G4 occupancy. G4 gains and losses were distributed throughout the genome, indicating that age-associated alterations in G4 occupancy involve both the accumulation of G4s at certain genomic *loci* and their depletion at others. The highest G4 enrichment was observed in the 72- and 73–year–old individuals, and other ages displayed variable occupancy, underscoring the complexity of age-related changes in G4 landscapes. (Fig. 4a, 4b). We next performed a regression analysis using individual age as a continuous variable to examine whether G4 enrichment at individual *loci* changed progressively with age **(Figure S5a)**. Most loci showed little or no progressive change with age, while a subset exhibited positive or negative age coefficients, reflecting variability across individuals rather than a uniform trend.

Analysis of G4 CUT&Tag signal around the TSS (± 2 kb) showed that promoter-proximal G4 enrichment was evident in all individual ages but varied in magnitude **(Figure S4a)**. Profiles from ages 22, 24, 32, and 34 displayed stronger and more sharply defined G4 enrichment near the TSS, while samples from 53 and 56 years showed lower enrichment, and individuals from 72 and 73 years had the weakest and most diffuse profiles. Extending this to a genome-wide view, violin plots of normalized G4 signal revealed relatively narrow distributions for ages 22, 24, 32, and 34, indicating more uniform G4 enrichment across peaks in these samples. In contrast, profiles from 53 and 56 years displayed broader distributions, and the widest spread was seen in 72 and 73 years, reflecting greater variability in G4 enrichment levels genome-wide **(Figure S4c)**.

Genomic annotation of G4 CUT&Tag peaks showed that G4 enrichment was distributed over multiple genomic compartments, with the largest proportion in intergenic and intronic regions (Fig. 3c). Comparison of differential peaks within age groups confirmed that most G4 peaks occurred in intergenic and intronic regions (Fig. 4c). We next examined the G4 content of these peaks, using the consensus G4-forming motif G_≥ 3_N_1–7_G_≥3_N_1–7_G_≥3_N_1–7_G_≥3_. Intron-associated G4s were more prominent in individuals aged 22, 24, 32, and 34 years, whereas intergenic canonical G4s were more prominent in samples aged 53, 56, 72, and 73 years. Promoter-associated canonical G4s that contain the standard G4 motifs were detected in all ages but represented a smaller fraction compared with intronic and intergenic sites (Fig. 4d, 4e). These results indicate that while canonical G4s follow the overall distribution of total G4 peaks, their relative representation across genomic categories differs with age. Although promoter-proximal peaks were well represented, a substantial fraction of G4 enrichment was located outside promoter regions, consistent with the ability of G4 structures to form within distal regulatory elements as well as within gene bodies. This distribution suggests that age-related variation in G4s may influence regulatory processes operating at both proximal and distal genomic sites.

We next examined the overlap of G4 peaks in all individual samples using an UpSet plot **(Figure S4d)**. This analysis revealed that many G4 peaks were specific to individual ages, with only a few overlaps between samples. Importantly, the largest shared set of peaks (roughly 1,200) was present across all samples, indicating a core group of G4 structures that are consistently detected in human astrocytes. The presence of this shared set is reassuring about the assay and likely reflects G4 formation at genomic regions where G-rich, single-stranded DNA is expected to occur, such as *loci* involved in transcription, repair, and replication. Beyond this core set, the largest age-specific overlap was between the 22- and 32-year samples. Most other intersections were smaller and represented peaks unique to one individual, indicating that G4 enrichment patterns vary greatly between individuals. Overall, these findings indicate that G4 landscapes in human astrocytes are heterogeneous yet show age-related accumulation, most pronounced at ages 72 and 73, with greater variability in younger adults, suggesting that G4 remodeling during adulthood may influence multiple layers of genome regulation in the aging brain.

#### Functional significance of age-associated G4 landscapes in human astrocytes

Age-associated G4 changes in astrocytes are heterogeneous genome-wide, with some *loci* showing reduced enrichment and others increased G4 occupancy. Even within the same chromosome, adjacent or nearby *loci* can display opposite patterns. For example, at the *GTF2A2 locus*, the G4 signal decreased progressively in samples from individuals aged 53, 56, 72, and 73 years compared with those from ages 22, 24, 32, and 34 (Fig. 4f). In contrast, the nearby *BNIP2 locus* showed higher G4 enrichment in the samples from 53, 56, 72, and 73 years relative to the individuals from 22, 24, 32, and 34 years (Fig. 4f). This illustrates that G4 remodeling with age can differ markedly even between closely positioned genomic regions.

A similar contrast is observed at the *PTPRG locus*. G4 enrichment was reduced from the samples aged 53, 56, 72, and 73 compared with the samples from 22, 24, 32, and 34 (Fig. 4g). In contrast, at the *NR_134500.1* and *NR_134499.1* loci, the G4 enrichment was consistently higher in individuals from 53, 56, 72, and 73 years (Fig. 4h). Additional loci from *XR_001745849.1* and *CEP78 loci* displayed increased G4 enrichment in the same ages, whereas RPTOR showed reduced G4 signal in the samples from 22, 24, 32, and 34 **(Figure S5b**–**5d)**. These examples illustrate that age-related changes in G4 occupancy are *locus*-specific rather than genome-wide. This heterogeneity suggests that G4 remodeling with age may influence different genomic regions in distinct ways, potentially altering their regulatory activity.

We next performed GO and KEGG pathway enrichment analyses to investigate the functional contexts of genomic regions showing altered G4 occupancy. Enrichment analysis of *loci* displaying reduced G4 occupancy revealed strong associations with immune and inflammatory regulation. Biological process terms included positive regulation of cytokine production, and processes linked to protein ubiquitination, chromatin organization, and fatty-acid metabolism (Fig. 4i). These *loci* were also enriched for molecular functions such as DNA transcription factor binding, RNA polymerase II-specific transcriptional regulation, and ubiquitin-protein transferase activity, suggesting that loss of G4s at these sites may impact transcriptional control of immune and stress-responsive genes **(Figure S5e)**. KEGG pathways connected to these *loci* included JAK-STAT signaling, AGE-RAGE signaling, NOD-like receptor signaling, and neutrophil extracellular trap formation, pointing to a broader attenuation of pro-inflammatory and immune signaling networks **(Figure S5g)**.

In contrast, *loci* with increased G4 occupancy were linked to cell-to-cell communication, signaling, and RNA metabolism. Biological processes enriched in this group comprise GPCR signaling, potassium ion transport, nervous system development, and RNA processing (Fig. 4j). Molecular function terms highlighted ion channel regulation, calcium channel activity, sequence-specific DNA binding, and GPCR activity, indicating potential modulation of excitability and transcriptional regulation in neuronal and signaling contexts **(Figure S5f)**. KEGG pathway enrichment further supported this shift, with significant representation of neuroactive ligand-receptor interaction, calcium and cAMP signaling, circadian entrainment, and synaptic transmission-related pathways **(Figure S5h)**. Overall, the functional enrichment analysis indicates that age-related remodeling of G4 structures in astrocytes is linked to shifts in key regulatory and metabolic pathways. These changes may contribute to altered transcriptional programs and cellular function during aging.

#### Integrative analysis of age-associated chromatin accessibility and G4 occupancy in human astrocytes

**Figure 5:**
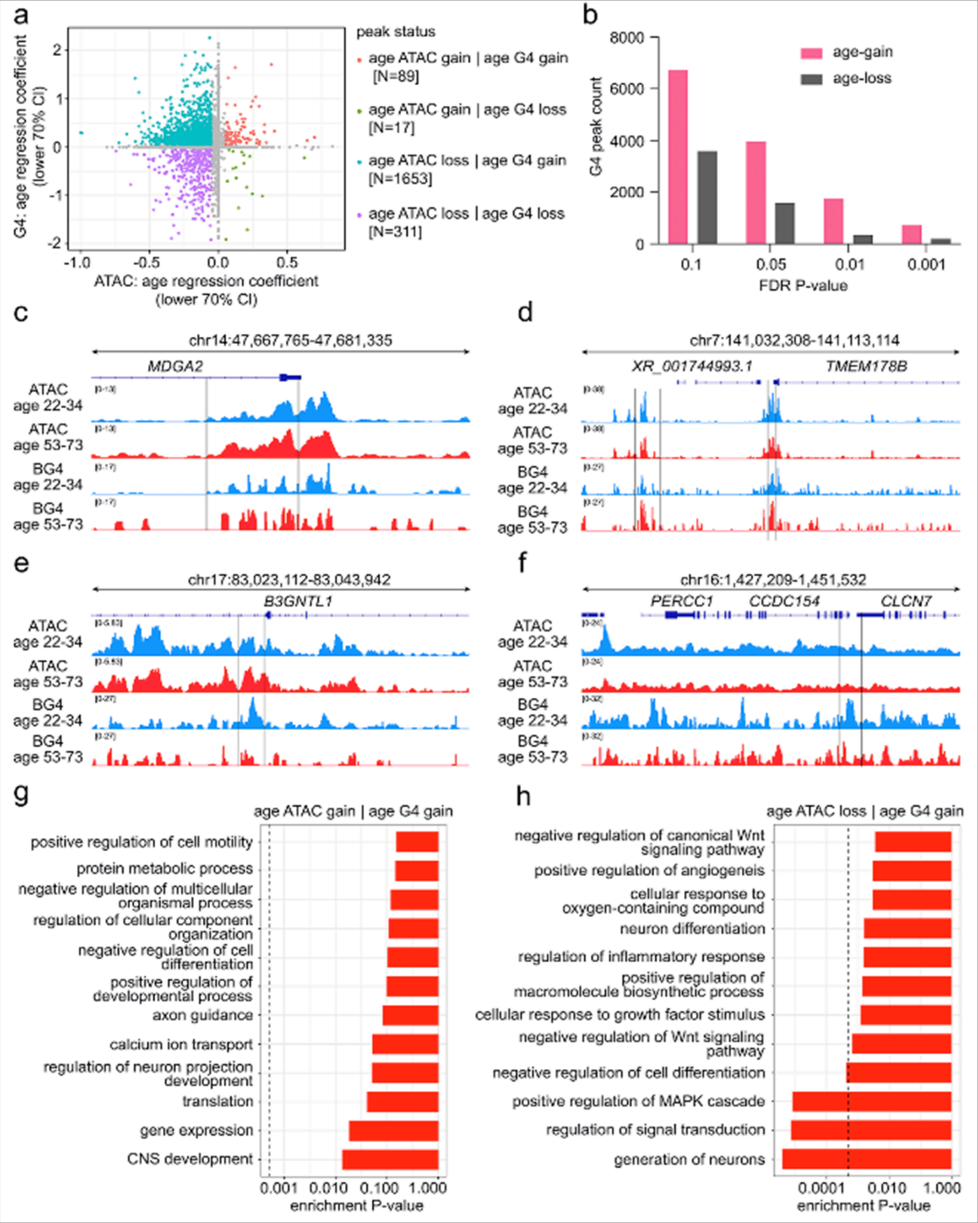
G4 structures persist and accumulate during aging at partially accessible chromatin regions in human astrocytes. **(a)** Joint regression analysis of age-associated changes in chromatin accessibility and G4 occupancy across shared genomic regions. The x-axis shows the lower bound of the 70% confidence interval (clamped at 0) for the age coefficient from ATAC-seq data, and the y-axis shows the corresponding value for G4 CUT&Tag. Each point represents a genomic peak with both ATAC-seq and G4 signal coverage. Peaks are categorized based on the directionality of change with age. Vertical and horizontal lines at the zero mark the threshold for age-related gain or loss in each modality. **(b)** Distribution of differentially enriched G4 peaks by false discovery rate (FDR) thresholds, stratified by direction of change with age. Bar plot shows the number of peaks with increased (age-gain, pink) or decreased (age-loss, gray) G4 occupancy in the 53, 56, 72, and 73 years, across a range of FDR cutoffs (0.1 to 0.001). **(c)** Genome browser (IGV) view showing ATAC-seq and BG4 signal at the *MDGA2 locus*, representative of regions with concurrent age-associated increase in chromatin accessibility and G4 signal (age ATAC gain | age G4 gain). The 22-, 24-, 32-, and 34-year samples are shown in blue, and the 53-, 56-, 72-, and 73-year samples are in red. **(d)** IGV plot at the *XR_001744993.1–TMEM178B locus*, representative of regions with increased G4 occupancy and reduced chromatin accessibility in the ages 53, 56, 72, and 73 years (age ATAC loss | age G4 gain). **(e)** IGV tracks showing ATAC-seq and BG4 peaks at the *B3GNTL1 locus*, representative of regions with increased chromatin accessibility and reduced G4 signal in the 53, 56, 72, and 73 years (age ATAC gain | age G4 loss). **(f)** Genome browser view at the *PERCC1–CCDC154–CLCN7 locus*, representing regions with reduced ATAC-seq signal and decreased G4 occupancy in the 53, 56, 72, and 73 years (age ATAC loss | age G4 loss). **(g)** GO Biological Process enrichment analysis of genes associated with genomic regions showing both increased chromatin accessibility and increased G4 signal (age ATAC gain | age G4 gain). **(h)** GO Biological Process enrichment analysis of genes linked to regions with decreased chromatin accessibility but increased G4 occupancy (age ATAC loss | age G4 gain).

G4s are believed to form preferentially within regions of open chromatin^43, 122^. Given our earlier observation that G4 changes over the age range were heterogeneous and *locus*-specific, we aimed to determine how these changes relate to age-associated alterations in chromatin accessibility. To address this, we performed a regression analysis using individual age as a continuous variable for both ATAC-seq and G4 CUT&Tag datasets, obtaining an age regression coefficient for each genomic *locus*. Positive coefficients indicated an increase with age, whereas negative coefficients indicated a decrease with age (Fig. 5a). By combining the directions of the ATAC and G4 coefficients, genomic regions were classified into four categories: ATAC gain + G4 gain, ATAC gain + G4 loss, ATAC loss + G4 gain, and ATAC loss + G4 loss. This classification enabled us to identify genomic *loci* where changes in accessibility and G4 occupancy occur in the same direction, as well as those where the two features change in opposite directions.

The scatter plot (Fig. 5a) visualizes this relationship by plotting the age-related regression coefficients for chromatin accessibility on the x-axis and those for G4 occupancy on the y-axis. Each point represents a genomic *locus* positioned according to the magnitude and direction of its change in both features across the age range. *Loci* in the upper-right quadrant (orange dots) show a coordinated increase in accessibility and G4 occupancy, whereas those in the lower-left quadrant (purple dots) show a coordinated decrease. The upper-left quadrant (blue dots) contains *loci* where accessibility decreases while G4 occupancy increases, and the lower-right quadrant contains *loci* where accessibility increases but G4 occupancy decreases. The spread of points across all four quadrants indicates that age-associated changes were not restricted to a single mode but included both coordinated and opposing shifts between chromatin accessibility and G4 occupancy.

We next quantified the number of G4 peaks showing either age-related gain or loss. Across all statistical cut-offs, *loci* with increased G4 enrichment were more frequent than those with reduced enrichment (Fig. 5b). Thus, while both gain and loss events occur, more genomic regions tend to acquire G4 enrichment with age than lose it, indicating that G4 accumulation is more common than depletion across the astrocyte genome during aging.

To illustrate these four categories of age-associated changes, we then examined representative genomic *loci* in Integrative Genomics Viewer (IGV) tracks, enabling direct visualization of how chromatin accessibility and G4 occupancy vary together or independently at specific sites. The ATAC gain + G4 gain category was exemplified by the *MDGA2 locus*, which showed a coordinated increase in both chromatin accessibility and G4 signal in the sample individuals from ages 53, 56, 72, and 73 years (Fig. 5c). In the ATAC loss + G4 gain category, the *XR_001744993.1* and *TMEM178B loci* demonstrated reduced chromatin accessibility in the individuals from 53, 56, 72, and 73 years, while G4 signal intensity increased over the same regions (Fig. 5d). The ATAC gain + G4 loss category was exemplified by the *B3GNTL1 locus*, where the chromatin accessibility was higher in samples from ages 53, 56, 72, and 73 years, and the G4 signal was diminished, suggesting that increased accessibility at this site does not necessarily coincide with stable or elevated G4 occupancy (Fig. 5e). Finally, for the ATAC loss + G4 loss category, the *PERC1–CCDC154–CLCN7* region exhibited concurrent reductions in both accessibility and G4 enrichment in the individuals from 53, 56, 72, and 73 years (Fig. 5f). These examples illustrate that the relationship between chromatin accessibility and G4 occupancy with age is highly *locus*-specific, encompassing coordinated gains or losses as well as opposing changes, depending on the genomic context.

To explore the potential functional consequences of these four peak-status categories, we performed GO and KEGG pathway enrichment analyses. This approach allowed us to link specific combinations of age-associated changes in chromatin accessibility and G4 occupancy to biological processes and pathways potentially influenced in astrocytes during aging. For *loci* showing ATAC gain + G4 gain, enrichment was prominent for processes related to neuronal function and cellular regulation, including positive regulation of cell motility, developmental processes, axon guidance, calcium ion transport, and CNS development (Fig. 5g). KEGG analysis further implicated signaling and synaptic pathways. These patterns suggest that coordinated increases in accessibility and G4 enrichment may be linked to transcriptional regulation of genes involved in neuronal connectivity, signaling, and adaptive cellular responses **(Figure S6c)**.

*Loci* showing ATAC gain + G4 loss were associated with processes including transcription by RNA polymerase II, calcium ion transport, chromatin organization, and TGFβ receptor signaling, alongside KEGG categories such as vascular smooth muscle contraction, dopaminergic and cholinergic synapses **(Figure S6a, S6d)**. This combination may indicate that while accessibility increases, the loss of G4 structures could alter regulatory stability at genes involved in transcriptional control, structural remodeling, and specialized signaling functions. *Loci* showing ATAC loss + G4 gain were enriched for developmental regulation, neuronal differentiation, angiogenesis, and inflammatory response regulation. KEGG pathways included Rap1 and Hippo signaling, PI3K-Akt signaling, calcium signaling, and pathways regulating stem cell pluripotency (Fig. 5h, **Figure S6e)**. These findings suggest that G4 accumulation in regions losing accessibility may occur at genes involved in long-term developmental and signaling programs, potentially reflecting compensatory or alternative regulatory states. Finally, *loci* showing ATAC loss + G4 loss were enriched for immune and stress-response processes, including inflammatory signaling, cytokine-mediated signaling, DNA repair, and autophagy. KEGG pathways encompassed immune response and infection-related categories such as JAK-STAT signaling and transcriptional misregulation in cancer **(Figure S6b, S6f)**. This dual loss pattern may reflect reduced regulatory engagement at immune- and repair-related genes during aging.

Overall, these analyses show that the relationship between chromatin accessibility and G4 occupancy during aging is functionally diverse and context-dependent, with each combination of changes linked to distinct biological processes and signaling pathways that influence different gene networks depending on the surrounding chromatin state.

#### DDX5 regulates transcription in human astrocytes

**Figure 6:**
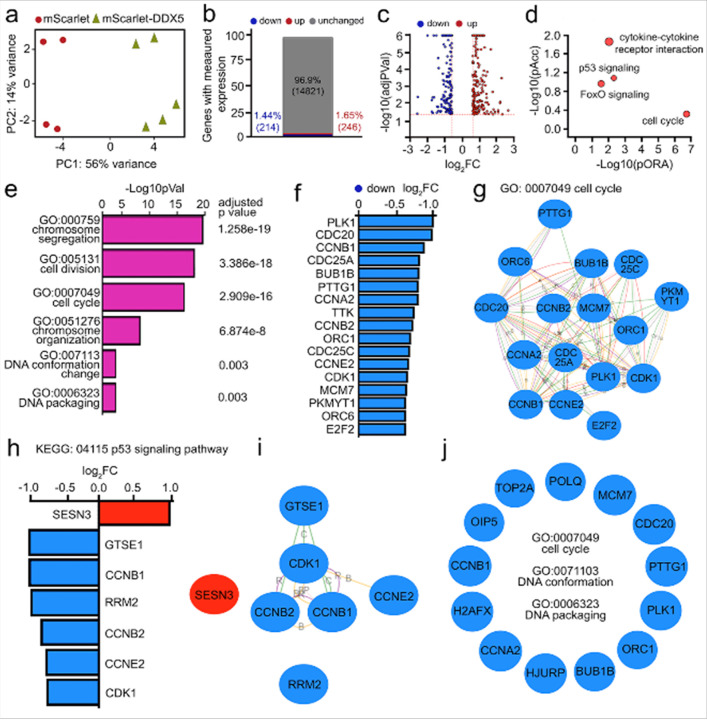
G4 helicase DDX5 regulates transcription in human astrocytes. **(a)** Principal component analysis (PCA) is used to define RNA-seq profiles. PCA of RNA-seq for 4 mScarlet–treated (red) and 5 mScarlet–DDX5 (green) treated in human astrocytes. Each dot represents one sample. The percentage values correspond to the percentage of total variance associated with each component. **(b)** 460 differentially expressed genes (DEGs) were identified out of a total of 14,821 genes with measured expression. Out of 460, 214 genes (1.44%) were downregulated and 246 (1.65%) were upregulated by DDX5. The thresholds used to select the DEGs were 0.6 for expression change and 0.05 for significance. **(c)** The volcano plot illustrates 460 DEGs. The significance is represented in terms of the negative log_10_ of the p-value, to plot more significant genes on the *y*-axis. The dotted lines represent the thresholds used to select the DEGs. The upregulated genes are shown in red dots, and the downregulated genes are shown in blue dots. **(d)** The pathway perturbation *versus* over-representation shows one of the most affected pathways by DDX5. Over-representation is shown on the *x*-axis (pORA), and the total pathway accumulation is shown on the *y*-axis (pAcc). Each pathway is represented by a single red dot, and the size of the dot is proportional to the size of the pathway it represents. The most affected pathway by DDX5 is the cell cycle pathway (KEGG: 04110), p=7.511 × 10^−6^. **(e)** Gene Ontology (GO) Biological Process enrichment analysis of differentially expressed genes following DDX5 overexpression. Enriched terms include chromosome segregation, cell division, chromatin organization, DNA packaging, and others. **(f)** An example of the cell cycle genes downregulated by DDX5. **(g)** A network of cell cycle regulatory genes is downregulated by DDX5. **(h)** An example of p53 genes affected by DDX5. **(i)** p53 hub genes are dysregulated by DDX5. Out of 7 DEGs, only one gene, SESN3, is upregulated, and the rest are downregulated. **(j)** An example of cell cycle (GO: 0007049), DNA conformation (GO: 0071103), and DNA packaging (GO:0006323) genes downregulated by DDX5.

Given the progressive, age-associated reduction in DDX5 expression together with focal accumulation of G4s and altered chromatin accessibility, we investigated the gene regulatory networks controlled by DDX5. We used human astrocytes from age 32 and overexpressed mScarlet-tagged DDX5 using lentiviral delivery, with mScarlet alone as a control. Subsequently, cells were subjected to RNA-sequencing analysis. Principal component analysis (PCA) of the RNA-sequencing results highlighted clear distinctions between the control mScarlet virus-infected samples and those infected with the mScarletDDX5 lentivirus. We identified a total of 460 differentially expressed genes (DEGs) out of a pool of 14,821 genes with measurable expression levels. Among these DEGs, 214 genes were upregulated (46%), while 246 genes were downregulated (54%). Notably, the upregulated genes tended to exhibit a more substantial fold change compared to the downregulated ones (Fig. 6a–6c). Pathway topologies, including genes and their directional interactions, were extracted from the KEGG database, and gene ontologies were sourced from the GO Consortium. This analysis identified 825 GO terms, 226 upstream regulators, and 29 disease categories that were significantly enriched in the DDX5-overexpressing samples. The most prominently affected pathways included the cell cycle, cellular senescence, aging, longevity, and the p53 signaling pathway. DDX5 was also found to regulate processes related to DNA topology, chromosome segregation, nucleosome assembly, and nuclear division (Fig. 6d, 6e).

#### DDX5 represses the cell cycle and p53 signaling pathways

One of the profoundly impacted pathways is the cell cycle (KEGG: 04110), with all 17 DEGs experiencing downregulation. Among these, Polo-like kinase (*PLK1*) stands out, as it plays a pivotal role in microtubule dynamics, DNA replication, chromosome dynamics, and p53 regulation^132–134^. DDX5 negatively regulates genes critical for G1/S and G2/M phase transitions, including *CCNB1*, *CDC25A*, and *CDK1*. Additionally, *CDC20* and *BUB1B*, key regulators of chromosome segregation and the spindle assembly checkpoint, were downregulated by DDX5. Furthermore, DDX5 negatively impacted the expression of *ORC1*, which is crucial for DNA replication initiation^135^, and *CDC25C*, governing cell division by directing cyclin B-bound CDC2 dephosphorylation and entry into mitosis^136^. DDX5 also diminished the expression of transcription factor *E2F2*, integral to cell cycle control and interactions with tumor suppressor proteins^137^ (Fig. 6f, 6g).

DDX5 modulates the expression of genes involved in the p53 signaling pathway (KEGG: 04115); six out of seven DEGs were downregulated. DDX5 upregulated Sestrin3 (*SEST3*), a stress-induced protein involved in reducing intracellular reactive oxygen species levels and implicated in blood glucose regulation, insulin resistance, and lipid storage in obesity^138^. In contrast, DDX5 downregulated *GTSE1*, which represses p53-mediated apoptosis, and mitotic regulators such as *CCNB1*, *CCNE2*, and *CDK1*^138, 139^ (Fig. 6h, 6i). The modulation of these genes highlights DDX5’s role in pathways governing cell proliferation and DNA damage response.

#### DDX5 influences chromatin organization, stress signaling, and genes associated with aging

DDX5 affected genes related to chromosome organization, DNA packaging (GO:0006323), DNA conformational changes (GO:0071103), nucleosome assembly (GO:0006334), and genome integrity (GO:0051276). 57 genes in these pathways were dysregulated. Notably, DDX5 upregulated *USP44*, which stabilizes the anaphase-promoting complex/cyclosome^140, 141^, while genes like *PKL1*, *POLOQ*, *TOP2A*, *UBE2C*, and *H2AX*, involved in chromatin remodeling and DNA structure, were downregulated. DDX5 also repressed *BRCA2*, *RAD54L*, and *MCM7*, which are critical for homologous recombination^142^ and replication^143, 144^, and *ERCC6*, a key DNA repair gene associated with chromosomal stability^145, 146^ (Fig. 6j).

Additionally, DDX5 negatively regulated *KIF4A*, an ATP-dependent microtubule-based motor protein involved in intracellular transport, chromosome integrity during mitosis, and central spindle organization before cytokinesis^147^. DDX5 exerted its influence on the FoxO signaling pathway (KEGG: 04068), resulting in the dysregulation of eight genes. Notably, upregulated genes like *FOXO1* and *BCL2L11* play pivotal roles in cell cycle regulation, DNA repair, apoptosis, and oxidative stress responses. Conversely, downregulated genes such as *PLK1*, *TNFSF10*, and *CCNB2* play diverse roles in cytokine-mediated apoptosis and cell cycle regulation **(Figure S5a)**.

DDX5 orchestrates the expression of genes associated with cellular processes in aging (GO:0007568). DDX5 upregulated 5 genes and downregulated 10 in this pathway. *CYP1A1*, encodes a member of the cytochrome P450 enzyme superfamily, crucial for drug metabolism and lipid synthesis^148^, experienced negative regulation by DDX5. In contrast, DDX5 positively affected *PRELP*, which maintains structural integrity by anchoring basement membranes to connective tissues and is linked to Hutchinson-Gilford progeria^149^. DDX5 also downregulated *DKK1*, a key inhibitor of Wnt signaling implicated in aging and neurological disorders such as Alzheimer’s disease^150, 151^. Concurrently, DDX5 negatively regulates *TYMS* (Thymidylate synthase), vital for DNA replication and repair, and considered a biomarker for aging and age-related diseases such as Alzheimer’s diseases^152^. DDX5’s intricate involvement underscores its pivotal role in cellular processes associated with aging, revealing its significant impact on gene functions.

#### DDX5 regulation of synaptic signaling and plasticity

The DDX5 impacts a set of 26 genes responsible for synaptic signaling (GO: 0099536) and synaptic plasticity (GO: 0048167). This cluster comprises 16 upregulated genes and 8 downregulated genes **(Figure S5b)**. Notably, DDX5 enhances the expression of *GRIK5*, a gene associated with glutamate-gated ion channels crucial for central nervous system excitatory neurotransmission^153^. DDX5 also upregulates *UNC13A*, a member of the UNC13 family, playing a role in neurotransmitter release^154^. Additionally, *PRRT2*, a transmembrane protein linked to episodic kinesigenic dyskinesia 1^155^, displays increased expression due to DDX5. Furthermore, DDX5 boosts the levels of Palmitoylated membrane protein 2 (*MPP2*), a member of the MAGUK family involved in cytoskeletal interactions and cell signaling^156^.

Conversely, DDX5 downregulates *NRGN*, a gene crucial for synaptic plasticity and often associated with neurological and mental disorders, particularly Alzheimer’s disease, where it serves as a reliable biomarker for synaptic dysfunction^157^. Taken together, we found that in human primary astrocytes, DDX5 modulates a number of crucial genes that are implicated in the physiology of synaptic transmission. These findings provide valuable insights into the specific astrocytic genes influenced by DDX5, emphasizing the crucial interplay between astrocytes and neurons in regulating synaptic function and contributing to a deeper understanding of the molecular basis of neurological disorders.

#### DDX5-regulated genes associated with diseases: Primary microcephaly, Fanconi anemia, and Bainbridge-Ropers Syndrome

Considering the myriad functions of DDX5 in regulating genes associated with crucial cellular processes, we analyzed disease pathways impacted by DDX5. The most prominently affected diseases include Primary microcephaly and Fanconi anemia. DDX5 negatively regulates genes associated with Primary microcephaly, a rare autosomal recessive neurodevelopmental disorder characterized by reduced brain volume and cognitive abnormalities^158^ **(Figure S5c)**. Notably, DDX5 downregulates *ASPM*, which regulates mitotic spindle function in neuroblasts during embryonic development^159^, and *CENPE*, a motor protein required for kinetochore microtubule capture and chromosome alignment during prometaphase^160^. *KNL1*, essential for kinetochore-microtubule attachments and accurate chromosome segregation^161^, and *WDR62*, implicated in cerebral cortical development and associated with microcephaly and cortical malformations, are also downregulated by DDX5^162^.

DDX5 also modulates genes associated with Fanconi anemia, a disorder characterized by bone marrow failure, chromosomal instability, and cancer susceptibility^163, 164^. Among the downregulated genes, *XRCC2* maintains chromosome stability and facilitates repair of DNA double-strand breaks *via* homologous recombination^165^. *FANCI*, a key component of the Fanconi anemia DNA damage signaling and repair pathway^166^, is also negatively regulated by DDX5. Together, these findings highlight the involvement of DDX5 in disease-associated pathways with critical roles in genome maintenance **(Figure S5d)**.

DDX5 upregulated the expression of *ASXL3*, a gene whose mutations are known to cause Bainbridge–Ropers syndrome, an *ASXL3*–associated developmental disorder characterized by severe intellectual disability, speech impairment, and distinctive craniofacial features^129, 167–170^. This finding suggests that DDX5 may influence pathways implicated in the pathogenesis of *ASXL3*-related disorders, potentially linking its regulatory activity to neurodevelopmental disease mechanisms.

#### DDX5 depletion alters the genomic distribution of G4 structures

Given the age-associated decline in the DDX5 expression in human astrocytes, we investigated whether acute depletion of DDX5 affects G4 abundance. Primary human astrocytes from a 24-year-old individual were transduced with lentiviral shRNA targeting DDX5 or a non-targeting shRNA control. DDX5 knockdown efficiency was confirmed by immunoassays and showed a marked reduction in DDX5 expression in DDX5-shRNA transduced cells compared with controls **(Figure S8a**–**c)**. To examine whether reduced DDX5 expression influenced G4 abundance, we performed G4 staining using N-TASQ and BG4: N-TASQ staining revealed an increase in G4 signal in knockdown cells compared to controls **(Figure S8d, e)**, whereas BG4 staining did not reach statistical significance, but it exhibited a consistent trend toward higher G4 levels in DDX5 KD astrocytes. **(Figure S8f, g)**.

**Figure 7:**
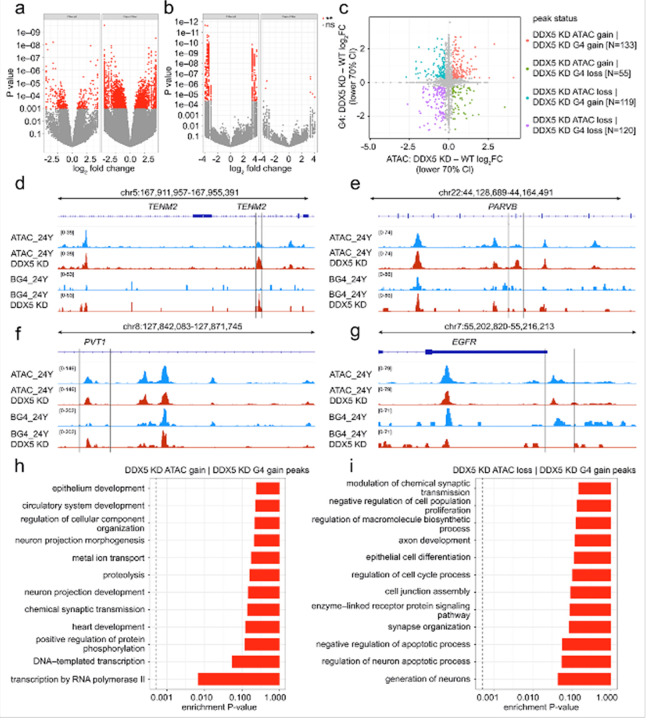
DDX5 depletion alters chromatin accessibility and G4 formation at gene regulatory *loci*. **(a) Volcano plot** showing promoter-level differential chromatin accessibility (ATAC-seq) between DDX5 knockdown (KD) and control astrocytes from a 24–year–old individual. Significantly altered peaks (FDR<0.05) are shown in red. **(b)** Volcano plot of promoter-level differential G4 occupancy in DDX5 KD and control astrocytes (FDR <0.05). **(c)** Concordance scatter plot comparing log_2_fold change in ATAC-seq (x-axis) and G4 CUT&Tag (y-axis) between DDX5 KD and control. Peaks are categorized into four groups: ATAC gain | G4 gain, ATAC gain | G4 loss, ATAC loss | G4 gain, ATAC loss | G4 loss. **(d)** Genome browser tracks illustrating representative *loci* with coordinated changes in chromatin accessibility and G4 occupancy upon DDX5 KD. Examples include TENM2 **(d),** PARVB **(e)**, PVT1 **(f)**, and EGFR **(g)** Blue = control; Red = DDX5 KD. **(h)** Gene Ontology (GO) enrichment analysis for genes associated with peaks showing concurrent ATAC gain and G4 gain in DDX5 KD astrocytes. **(i)** GO enrichment for genes associated with peaks showing ATAC loss and G4 gain in DDX5 KD astrocytes. Bars represent P values for significantly enriched biological processes. Both gene sets show limited enrichment, indicating that these categories do not map strongly onto defined biological pathways.

To further assess whether DDX5 depletion alters chromatin accessibility and G4 occupancy, we performed genome-wide ATAC-seq and G4 CUT&Tag profiling in control and DDX5-knockdown astrocytes. Differential analysis of ATAC-seq data revealed a limited number of peaks with significant changes in chromatin accessibility following DDX5 knockdown (Fig. 7a). Most genomic regions retained accessibility levels comparable to control cells, indicating that acute DDX5 depletion does not broadly alter chromatin structure **(Figure S2a, c, d)**. In contrast, G4 CUT&Tag profiling identified focal G4 accumulation at a subset of genomic *loci* (Fig. 7b), including both gains and losses at discrete sites **(Figure S4a, c, d)**.

Functional enrichment of differentially accessible chromatin regions revealed significant associations with chromatin remodeling, neurogenesis, DNA repair, and angiogenesis, as well as signaling cascades including PI3K-Akt, apoptosis, and AGE-RAGE signaling **(Figure S9a–S9d)**. Regions showing focal G4 accumulation were linked to angiogenesis, cell adhesion, cytoskeletal organization, calcium ion transport, and synaptic signaling, with KEGG pathways implicating JAK–STAT, MAPK, circadian entrainment, and glutamatergic synapse regulation **(Figure S9e–h)**, suggesting that focal G4 changes may occur within functionally relevant regulatory contexts.

Regression analysis integrating ATAC-seq and G4 CUT&Tag profiles in DDX5-depleted astrocytes revealed that most genomic regions grouped toward the central range of the distribution with a smaller fraction of regions exhibiting concurrent shifts in both chromatin accessibility and G4 signal, while other regions displayed changes in chromatin accessibility without corresponding differences in G4 signal, or vice versa (Fig. 7c). To illustrate these patterns, we examined representative *loci* from each of the four regression categories (Fig. 7d–g. At *TENM2*, both accessibility and G4 signal increased (ATAC gain + G4 gain), whereas *PARVB* showed increased accessibility but reduced G4 occupancy (ATAC gain + G4 loss). In contrast, *PVT1* displayed reduced accessibility with increased G4 enrichment (ATAC loss + G4 gain), and *EGFR* exhibited decreases in both (Fig. 7g) (ATAC loss | G4 loss). These examples demonstrate that DDX5 depletion can produce *locus*-specific changes in accessibility and G4 enrichment.

Functional enrichment analysis of these regression categories revealed distinct biological associations. ATAC gain + G4 gain was linked to neuronal development and synaptic signaling; ATAC loss + G4 gain to cell cycle and axon development (Fig. 7h, i). ATAC gain + G4 loss to autophagy and DNA metabolic processes; and ATAC loss + G4 loss to vasculature development and DNA damage repair **(Figure S10a, b)**. KEGG pathways implicated across categories included MAPK, PI3K–Akt, mTOR, and FoxO signaling, as well as neurodegeneration-related pathways **(Figure S10c–f)**. Taken together, these results demonstrate that while acute DDX5 depletion does not induce widespread alterations in chromatin accessibility, it is associated with focal accumulation of G4 structures at selected genomic *loci*, highlighting a targeted role for DDX5 in maintaining G4 homeostasis and chromatin organization.

## Discussion

Understanding how chromatin organization and G4 biology change with age is critical for uncovering molecular mechanisms that contribute to cellular aging. Astrocytes are central to brain function, influencing synaptic maintenance, neuronal metabolism, and neuroinflammatory responses, and their molecular state directly impacts neuronal health. To investigate these mechanisms in a physiologically relevant context, we established primary human astrocyte cultures from cortical resections of individuals spanning the adult lifespan (22–73 years). Unlike immortalized astrocyte lines, which acquire non-physiological chromatin states during continuous passaging, or induced pluripotent stem cell–derived astrocytes, which lose age-associated molecular and structural features through epigenetic “rejuvenation” during reprogramming, our model system preserves the individual-specific chromatin landscape and molecular features across the age spectrum. Importantly, because these resections were made from epileptic patients, analysis of our datasets revealed no enrichment of epilepsy-related pathways. This preservation is essential for studying age-dependent changes in genome architecture and G4 dynamics.

These astrocytes exhibit molecular hallmarks associated with cellular aging, and we discovered an accumulation of G4 structures in the oldest cells, accompanied by a concomitant decline in expression of the G4-resolving helicase DDX5. This imbalance favors the formation and persistence of G4 structures at specific genomic sites, where their prolonged presence can alter local chromatin folding, restrict access of regulatory proteins, interfere with transcriptional activity, and trigger genetic instability^49, 54^. Over time, such unresolved G4s could reconfigure the landscape of the genome, thereby influencing the stability of gene expression programs in astrocytes during aging.

To determine whether the age-associated shift in G4 homeostasis and decline in DDX5 expression were accompanied by broader alterations in genome architecture, we performed ATAC-seq on these astrocytes. The analysis revealed a clear age-dependent decline in chromatin accessibility across the genome, with the most pronounced losses occurring at promoter-proximal regions. Heatmap clustering of the top differentially accessible peaks showed that samples separated according to sample age, with accessibility loss following a coordinated, age-linked pattern rather than stochastic variation. Volcano plots further confirmed that the number and magnitude of regions showing reduced accessibility far exceeded those with gains, indicating that chromatin closure was the dominant feature between age comparisons. These observations are consistent with independent studies, including evidence of global open-chromatin loss in glial populations^171^ and meta-analysis of aging datasets showing a roughly 2:1 imbalance of accessibility losses versus gains in astrocytes^172^.

Transcription start site–centered profiles revealed reduced accessibility flanking promoters, suggesting a decline in the permissive chromatin environment required for efficient transcription initiation. Violin plots demonstrated that this reduction was consistent in all samples within the same age range, while UpSet plot analysis showed that many of the regions losing accessibility were shared among multiple individuals, pointing to a core set of regulatory *loci* undergoing age-dependent compaction. This coordinated loss of promoter accessibility suggests that regulatory regions in astrocytes become progressively less permissive with advancing age. Reduced accessibility at promoters can limit the recruitment of transcription factors and co-activators, thereby constraining the activation of gene programs required for stress adaptation, synaptic regulation, and metabolic support. Because astrocytes play essential roles in maintaining neuronal function, such widespread restriction of promoter function may contribute to diminished cellular responsiveness and impaired support for neuronal networks during brain aging. Notably, this pattern contrasts with the prevailing view from studies in other cell types, where aging is often associated with global chromatin relaxation^1, 8, 12, 173^. The observation that astrocytes instead exhibit promoter-focused compaction suggests that chromatin aging is highly celltype specific, warranting further examination of the molecular programs that drive this distinct trajectory.

Several interconnected molecular processes could explain the widespread reduction in chromatin accessibility observed in aging astrocytes. Among these, changes in histone modification and DNA methylation patterns are well-documented features of human aging^12, 15^. Loss of activating marks such as H3K27ac and H3K4me3, together with enrichment of repressive marks like H3K9me3 and H3K27me3, can promote chromatin compaction at regulatory regions^12, 13^. In some contexts, increased acetylation has been reported at stress-responsive *loci*, enabling localized chromatin opening. In astrocytes, the overall balance of these epigenetic shifts may tilt toward reduced accessibility across the genome, consistent with the promoter-level restriction revealed by our ATAC-seq analysis. Chromatin remodeling associated with persistent DNA damage may further reinforce this state, as repair-associated remodeling complexes can establish compact heterochromatin around damage *foci*. Alterations in architectural proteins such as CTCF and cohesin, which maintain promoter-enhancer loops and chromatin boundaries, may weaken regulatory contacts and favor tighter nucleosome packing^174–177^. Chronic inflammatory, oxidative, and metabolic stress in the aging brain can also drive adaptive chromatin programs that restrict promoter accessibility to minimize transcriptional noise and protect genome stability, albeit at the expense of reduced responsiveness to physiological cues. Together, these factors likely work in concert to shape the more restrictive chromatin state we observe in aging astrocytes and could influence the formation, stability, and genomic localization of G4 structures.

G4 CUT&Tag profiling revealed a more complex pattern of age-associated changes than the clear, progressive trend seen in our ATAC-seq data. Among all individuals, the G4 signal did not segregate by age, but the oldest individuals aged 72 and 73 showed a pronounced G4 enrichment at specific genomic sites, consistent with the strong G4 increase detected by N-TASQ and BG4 staining. Annotation of differential peaks showed that these changes were not randomly distributed throughout the genome. G4 peaks were frequently located in intronic, intergenic, promoter-proximal regions, but their relative representation shifted with age, with a clear pattern of increased representation in intergenic and promoter regions and a lower proportion in introns. This indicates a strong age-associated shift in G4 state towards increased occupancy, preferentially involving specific regulatory and non-coding domains rather than affecting all genomic compartments equally.

Integrating G4 CUT&Tag with ATAC-seq data provided a framework to interpret how G4 remodeling relates to chromatin state in aging astrocytes. The most prominent pattern was G4 enrichment at *loci* with reduced chromatin accessibility, suggesting that G4 structures may persist or accumulate within compact chromatin domains and potentially reinforce a repressive state. In contrast, regions that gained accessibility often showed a reduction in the G4 signal. While G4 formation is generally favored in open chromatin where the underlying motifs are exposed, the relationship between accessibility and steady-state G4 occupancy is more complex. In highly accessible *loci* undergoing active transcription, G4 structures may be transient, as polymerase passage and helicase activity can rapidly resolve them to prevent transcriptional stalling. During aging, shifts in transcriptional programs, altered chromatin-remodeling activity, and reduced expression of G4-resolving helicases such as DDX5 may further influence this balance, allowing persistent G4s to accumulate in compact chromatin while active transcription in open regions continues to favor their resolution. Importantly, a reduction in ATAC signal does not necessarily equate to a complete loss of euchromatin. One possibility is that G4s themselves may act to stabilize euchromatin by preventing nucleosome deposition or blocking the recruitment of chromatin remodelers. In such a scenario, *loci* that show increased G4 formation but reduced chromatin accessibility may represent regions where G4 structures hinder complete heterochromatin formation, resulting in the G4 gain | ATAC loss signature observed in some subsets of cells. Regions showing gains in both accessibility and G4s may reflect active domains where G4s form without preventing transcription, whereas concurrent loss of accessibility and G4s likely represents broader chromatin remodeling away from both open chromatin and G4 structure formation. Together, these patterns indicate that G4 redistribution during aging is closely tied to chromatin remodeling, reinforcing repression at some *loci* while accompanying activation at others.

Given that reduced expression of DDX5 may contribute to this remodeling, we next examined the transcriptional programs directly regulated by DDX5 in human astrocytes to better understand how changes in its activity could shape the interplay between chromatin accessibility, G4 occupancy, and gene expression. Overexpression of DDX5 in astrocytes from a 32-year-old individual revealed coordinated changes in transcriptional programs related to genome stability, stress adaptation, and astrocyte-neuron communication. Genes controlling cell-cycle progression and DNA replication were downregulated, while pathways involved in stress response, chromatin organization, and synaptic signaling were upregulated. These patterns align with the features identified in our ATAC-seq and G4 CUT&Tag datasets, where promoter accessibility shifts and focal G4 persistence were prominent in aging-associated remodeling. This correspondence suggests that DDX5 supports the activity of specific genes by resolving G4 structures and maintaining promoter accessibility, where both are critical for transcription. In doing so, DDX5 may help regulate genome integrity and sustain the functional capacity of astrocytes to support neurons over time. Reduced DDX5 levels with age could compromise this regulatory role, making it harder for astrocytes to activate protective and adaptive gene programs, thereby contributing to the progressive decline in astrocyte function during brain aging.

Acute depletion of DDX5 in astrocytes resulted in localized changes in chromatin accessibility and G4 occupancy, without inducing genome-wide shifts observed during aging. Several factors may contribute to this difference. First, the decline of DDX5 with aging is gradual and sustained over decades, allowing downstream effects such as epigenetic reprogramming, chromatin remodeling, and shifts in transcriptional programs to fully establish and exert physiologically relevant effects. Acute depletion of DDX5 does not provide the temporal scale required for these processes to occur. Second, astrocytes under baseline physiological conditions could maintain high expression of other G4-resolving helicases, such as DHX36, WRN, and BLM, which may functionally compensate for the loss of DDX5 and limit global G4 stabilization. Third, the chromatin landscape in astrocytes is inherently more open and dynamic, which can favor efficient resolution of G4 structures where helicases and transcriptional machinery can access these sites more readily, preventing G4s from becoming stabilized. Finally, it is likely that DDX5 loss acts in concert with other age-associated molecular changes, such as cumulative alterations in chromatin modifiers, transcription factors, and DNA repair pathways, to promote persistent G4 accumulation and large-scale remodeling. Without these additional, co-occurring molecular shifts, acute depletion alone may be insufficient to recapitulate the broader alterations seen during aging.

**Figure 8:**
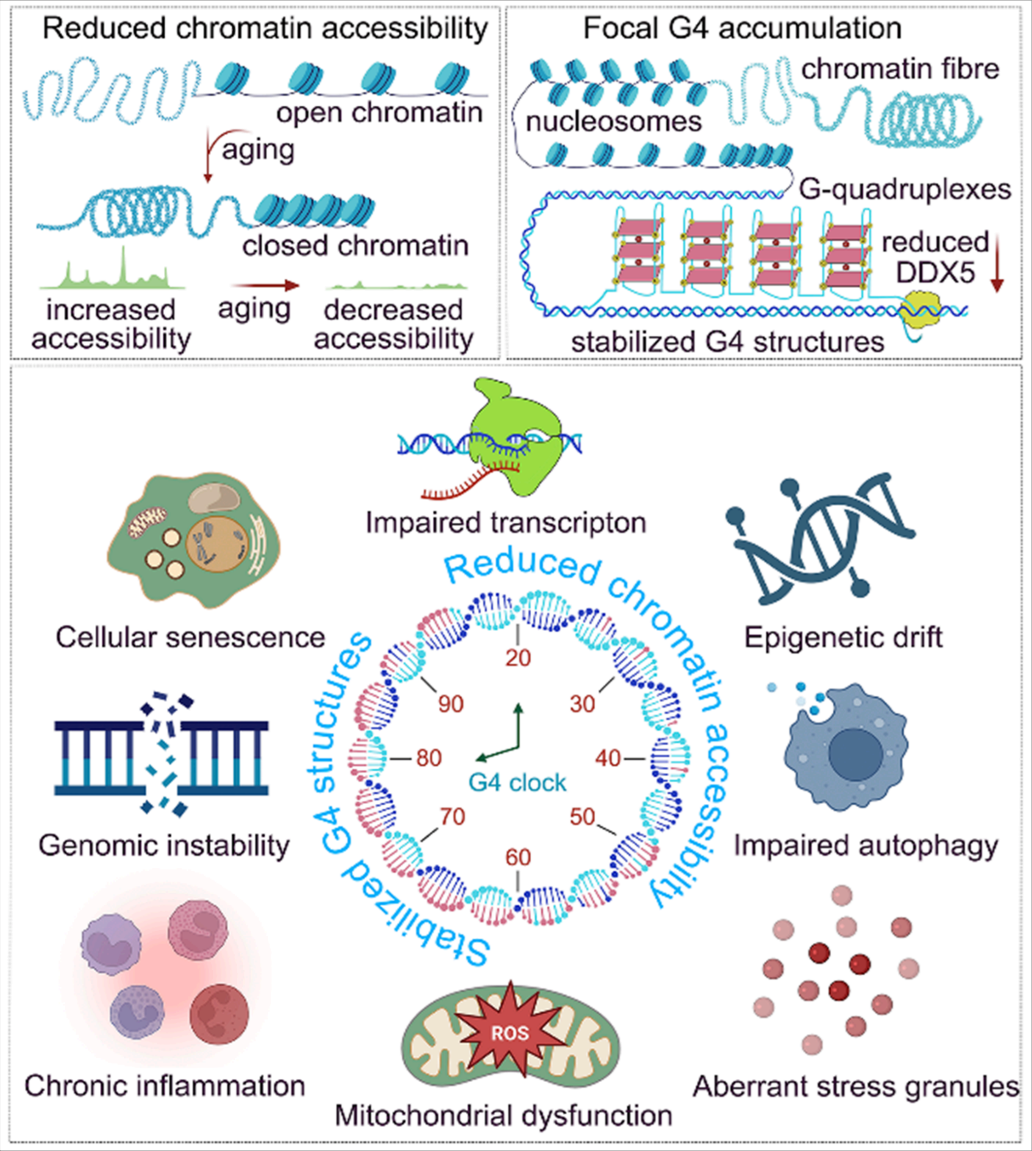
Model of age-associated chromatin remodeling driven by reduced accessibility and focal G4 accumulation. In our model of aging, chromatin progressively loses accessibility, leading to widespread closing of regulatory regions (top left). Focal stabilization of G4 structures was detected at specific genomic loci in older astrocytes, accompanied by reduced expression of G4 helicase DDX5 (top right). These two age-associated changes, loss of chromatin accessibility and accumulation of G4 structures, define a regulatory signature we term the “G4 clock” (center), which captures the combined epigenetic drift of the aging genome. The G4 clock represents a shift from dynamic chromatin regulation toward stabilized, less accessible states that impair transcriptional output. This altered chromatin landscape is associated with multiple hallmarks of aging, including cellular senescence, genomic instability, chronic inflammation, mitochondrial dysfunction with ROS accumulation, impaired autophagy, epigenetic drift, and aberrant stress granule formation (bottom). This integrated model positions the G4 clock as a conceptual framework linking helicase dysfunction, reduced chromatin accessibility, and G4 accumulation to transcriptional dysregulation and multiple hallmarks of aging.

Our integrative analysis of chromatin accessibility, G4 mapping, and DDX5 transcriptional profiling demonstrates that aging astrocytes undergo selective remodeling of both chromatin organization and G4 architecture (Fig. 8). Within this framework, DDX5 emerges as an important component of the molecular machinery that regulates the interplay between G4 formation and resolution and maintains accessibility at specific regulatory *loci*. Importantly, these effects arise within a broader landscape of age-associated alterations, where declining DDX5 function likely acts in synergy with other molecular changes to influence G4 dynamics and chromatin states. (Fig. 8). Given the structural diversity and regulatory complexity of G4s, their persistence or resolution may be determined by the combined influence of helicase activity, chromatin context, and long-term physiological stress. We propose that these combined influences give rise to an age-dependent regulatory signature, which we describe as the “G4 clock,” that integrates chromatin closure, helicase dysfunction, and focal G4 accumulation as interdependent processes shaping transcriptional control and cellular homeostasis during aging.

## Conclusion

Genome-wide profiling of chromatin accessibility and G4 landscape in aging astrocytes reveals discrete regulatory regions that may serve as leverage points for intervention. G4 structures are inherently dynamic, and their resolution can be modulated pharmacologically by small molecules, making them a potentially tractable layer of genome regulation^114, 115, 178–181^. Within the geroscience framework, such strategies could help modify age-related chromatin remodeling and preserve cellular resilience. The links between altered G4 states and molecular pathways disrupted in neurodegenerative disease underscore their broader therapeutic relevance^182^. Advances in G4 biology techniques, such as G4access, which enables high-resolution, antibody-independent G4 mapping, now provide the additional precision needed to score and chart G4 structures across physiological and pathological contexts^43^. Applying these tools in aging models will clarify where targeted modulation of G4 states could be most effective in sustaining transcriptional adaptability and cellular function over time.

## Methods

### Chemicals and antibodies.

Antibodies against Aquaporin were from Sigma-Aldrich (catalog No: HPA014784); Vimentin from Cell Signaling Technology (catalog No: 3932S); EAAT2 from Santa Cruz Biotechnology (catalog No: SC-365634); S100β from Abcam (catalog No: EP1576Y); GFAP from Novus Biologicals (catalog No: NBP1–05198); p16 from Santa Cruz Biotechnology (catalog No: SC-1661); p21 from Cell Signaling Technology (catalog No: 2947T); p53 from Novus Biologicals (catalog No: NB200–103); DDX5 from Cell Signaling Technology (catalog No: D15E10); DIRAS1 from Novus Biologicals (catalog No: NBP1–58935); BG4 from Sigma-Aldrich (catalog No: MABE917 (antibody developed by Balasubramanian lab)). All secondary antibodies used were from Life Technologies, including Anti-rabbit Alexa Fluor 488-labeled (catalog No. A11008), anti-mouse 488-labeled (catalog No. A11001), and anti-chicken Alexa Fluor 647-labeled (catalog No. A21449). mScarlet, mScarlet-DDX5 viruses were custom-designed and procured from VectorBuilder.

### Isolation and culture of primary human astrocytes.

In this study, we collected epileptic brain tissue resections from the frontal lobes of patients aged 22-M, 24-M, 32-M, 34-M, 53-M, 56-F, 72-M, and 73-F, maintaining sterility throughout the process. All the patients had no known additional pathological conditions or co-morbidities, and ethical standards, including obtaining informed consent, were observed throughout the handling of human tissue samples. The brain samples were subjected to a 30-second sterilization step using a 20% Betadine solution, followed by thorough rinsing with 1X PBS. Subsequently, the tissue was finely diced into 1mm-sized pieces on a sterile petri dish, and these explants were then transferred to PDL-coated plates and air-dried for 5 minutes. The explants were cultured in a DMEM growth medium (catalog No. SH30261.01, Cytiva Life Sciences) supplemented with 10% FBS (catalog No. F00926, Sigma-Aldrich), as well as penicillin and streptomycin (catalog No. SV30010, Cytiva Life Sciences). After culturing them for 2–3 weeks, astrocytes were isolated from the explants and continued their culture in DMEM growth medium enriched with recombinant human TGF-α (catalog No. 239-A, Bio-techne R&D systems), human NRG1-β1 (catalog No. 396-HB, Bio-techne R&D systems) and human insulin (catalog No. 12585014, ThermoFisher Scientific) growth factors, promoting their growth until 3–4 passages.

### ATAC-seq – Cell preparation and nuclei isolation.

Primary human astrocyte cultures were thawed at 37°C in a water bath until a small ice crystal remained. Cells were immediately diluted in RPMI supplemented with 10% FBS and centrifuged at 300 × g for 5 minutes. Pellets were washed twice in PBS containing 0.04% BSA and passed through a 40 μm cell strainer to remove debris. Cell viability and counts were assessed using a hemocytometer prior to nuclei isolation. For lysis, cells were resuspended in 100 μL of chilled lysis buffer [10 mM Tris-HCl (pH 7.5), 10 mM NaCl, 3 mM MgCl_2_, 0.1% Tween-20, 0.1% Non-idet P-40 substitute, 0.01% digitonin, 1% BSA, 1 mM DTT, and 1× protease inhibitors] and incubated on ice for 3 minutes. Nuclei were washed three times in 1 mL of wash buffer [10 mM Tris-HCl (pH 7.5), 10 mM NaCl, 3 mM MgCl_2_, 0.1% Tween-20, 1% BSA, 1 mM DTT, 1× protease inhibitors] at 500 × g for 5 minutes at 4°C. Nuclei were resuspended in PBS + 0.04% BSA, counted, and typically yielded ~ 4 × 10^6^ nuclei per sample.

### Tagmentation and library preparation.

Fifty thousand nuclei were resuspended in 12.5 μL of 1× nuclei buffer and incubated with pA-Tn5 transposase preloaded with sequencing adapters. Tagmentation was performed in a thermocycler at 37°C for 30 minutes. Tagmented DNA was purified using the Zymo DNA Clean & Concentrator kit (Zymo Research) and eluted in 21 μL of nuclease-free water. Libraries were amplified in 50 μL reactions using NEB-Next High-Fidelity 2× PCR Master Mix (NEB, Cat# M0541) and 2.5 μL each of 10 μM indexed i5 and i7 primers. PCR cycling conditions were: 72°C for 5 min, 98°C for 30 s, followed by 12 cycles of 98°C for 10 s, 63°C for 30 s, and 72°C for 1 min, with a final extension at 72°C for 1 min. Amplified libraries were cleaned with 0.75× SPRI-select beads (0.75x) with two 80% ethanol washes and eluted in 20 μL water.

### G4 CUT&Tag – Cell crosslinking and nuclei isolation.

Astrocyte cultures were crosslinked by adding 31.25 μL of 16% formaldehyde to 1 mL of cell suspension in PBS + 0.04% BSA to reach a final concentration of 0.5%. Samples were incubated on ice for 5 minutes and quenched with 50 μL of 2.5 M glycine. Cells were pelleted at 300 × g for 5 minutes and washed once with PBS. Cells were lysed in chilled lysis buffer [10 mM Tris-HCl (pH 7.4), 10 mM NaCl, 3 mM MgCl_2_, 0.1% Tween-20, 0.1% NP-40 substitute, 0.01% digitonin, 1% BSA, 1 mM DTT, and 1× protease inhibitors] using 200 μL per large sample or 100 μL for small samples. Lysis was performed on ice for 5 minutes, followed by two washes with 1 mL wash buffer [same composition as above without NP-40 substitute]. Nuclei were resuspended in PBS + 0.04% BSA and counted. 100,000 nuclei were aliquoted per CUT&Tag reaction.

### Antibody binding and tagmentation.

Nuclei were resuspended in 70–90 μL of incubation buffer, antibodies, and pAG-Tn5 added. The following antibodies and enzymes were added per reaction: Primary antibody: G4 scFv (Millipore MABE917, 0.25 mg/mL), 1.03 μL; Secondary antibody: Anti-FLAG M2 (Millipore F1804, 1 mg/mL), 1.48 μL; pAG-Tn5 transposome (EpiCypher, Cat# 15–1025-FT002, 3.94 μM dimer), 2.53 μL. Samples were incubated overnight at 4°C with gentle rotation. Tagmentation was triggered by adding 4 μL of 250 mM MgCl_2_ and incubating at 37°C for 1 hour at 550 rpm. The reaction was quenched with 20 μL of 80 mM EDTA. DNA was purified using the Zymo DNA Clean & Concentrator5 kit and eluted in 20 μL of nuclease-free water.

### Library amplification.

PCR reactions (50 μL) included 25 μL NEBNext High-Fidelity 2× Master Mix and 2.5 μL of 10 μM indexed i7/i5 primers. Thermal cycling was done at 72°C for 5 min; 98°C for 30 s; 15 cycles of 98°C for 10 s, 63°C for 30 s, 72°C for 1 min; final extension at 72°C for 2 min. SPRIselect beads (0.75×) were used for purification, and libraries were eluted in 10 μL of water. DNA concentration was measured by Qubit, and libraries were diluted to 1 ng/μL for TapeStation QC.

### Quality control and bioinformatics analysis.

Quality control metrics confirmed high-quality data generation from astrocyte cultures for both ATAC-seq and G4 CUT&Tag assays. ATAC-seq libraries exhibited alignment rates exceeding 95%, with a fragment-in-peak (FRiP) score of approximately 50% and ~ 12 million unique fragments per sample, consistent with high library complexity and effective profiling of accessible chromatin. In contrast, G4 CUT&Tag libraries showed lower alignment rates (~ 30%) and FRiP scores (~ 11%), as expected for a challenging target such as G4-DNA. Despite this, library complexity remained acceptable, with post-deduplication fragment counts ranging from 1 to 6 million. TapeStation profiles confirmed successful library construction, albeit with slightly reduced concentrations compared to typical ATAC-seq yields. Mitochondrial DNA contamination was minimal, accounting for < 3% of reads in ATAC-seq and < 10% in G4 CUT&Tag. Transcription start site (TSS) enrichment scores for ATAC-seq met established quality thresholds. Peak calling using MACS2 (narrow peak mode) identified approximately 322,533 peaks for ATAC-seq and 49,770 peaks for G4 CUT&Tag data in astrocytes.

### Analysis pipelines.

Sequencing libraries from astrocyte samples were generated on an Element AVITI platform using 2 × 150 bp paired-end reads. Raw sequencing data were assessed using FastQC to evaluate per-base sequence quality, adapter contamination, sequence duplication rates, and overrepresented sequences. High-quality reads were aligned to the human reference genome (GRCh38) using BWA with default parameters, and coordinate-sorted BAM files were produced. PCR duplicates were removed, and unique fragment pairs were extracted to generate BED files, with each entry corresponding to a single start–end fragment. These fragments were further processed into 50 bp cutsite representations centered at each end to facilitate downstream analysis. Peak calling was performed using MACS2 in paired-end mode with the --nomodel flag and a 75 bp fragment extension to account for the footprint of Tn5 tagmentation. This approach was applied uniformly to both ATAC-seq and G4 CUT&Tag datasets.

### Peak annotation.

Peak annotation was performed using PyRanges, referencing a consolidated transcript model based on MANE v1.4 (RefSeq, hg38). Promoter regions were defined as the interval spanning − 2.5 kb upstream to + 250 bp downstream of each annotated transcription start site (TSS). Additional genomic features included first exons, all exons, 5′ untranslated regions (5′ UTRs), 3′ UTRs, introns, and downstream regions extending up to 3 kb beyond the annotated transcript end. Each peak was assigned a single annotation label according to a predefined hierarchical scheme prioritizing functionally proximal features in the following order: first exon > promoter > exon > 5′ UTR > 3′ UTR > intron > downstream > intergenic. In parallel, the distance to the nearest transcript boundary was computed in both upstream and downstream directions, and corresponding gene names were appended as tss_distance_upstream, tss_distance_downstream, gene_upstream, and gene_downstream for each peak.

### G4 canonical site annotation.

To identify canonical G4 motifs within accessible chromatin, all ATAC-seq peaks were first expanded by 15 base pairs on each end using a genomic interval slop operation to capture adjacent guanine-rich regions. The resulting extended intervals were used to extract the corresponding DNA sequences from the GRCh38 reference genome. These sequences were scanned using a regular expression pattern designed to detect canonical G4 motifs, defined as four runs of at least three consecutive guanines separated by one to seven arbitrary nucleotides. The annotation pattern used was: G{3,}[ATGC]{1,7}G{3,}[ATGC]{1,7}G{3,}[ATGC]{1,7}G{3,}. The Peaks containing at least one match to this motif were annotated as canonical G4 sites.

### Integration of normalized coverage metrics.

Normalized coverage metrics were computed to assess signal strength and variability across all peaks. For each assay (ATAC-seq and G4 CUT&Tag), per-peak coverage summaries were obtained from tab-delimited files generated by a custom coverage-intersection pipeline. Three key metrics were extracted: (i) max_depth, the raw maximum read depth within the peak; (ii) avg_depth, the mean per-base coverage; and (iii) norm_depth, calculated by scaling the maximum depth using a global normalization factor defined as the reciprocal of the 20% trimmed-mean coverage over randomly selected non-peak genomic regions. The norm_depth values across all replicates were compiled into matrices (atac_nrm and g4q_nrm), from which the mean normalized depth, standard deviation, and log-space standard deviation [exp(sd(log(1e–4 + norm_depth)))] were computed for each peak. These metrics captured both absolute signal magnitude and relative variability, enabling reliable peak evaluation. To further improve data quality, peaks overlapping ENCODE blacklist regions (hg38) were excluded, and only those located on autosomes or chromosome X were retained for downstream analyses.

### G-quadruplex peak refinement.

To improve the specificity of G4 CUT&Tag peak calls and reduce background from low-confidence regions, a multi-step refinement strategy was applied. First, peaks with a mean normalized signal greater than 0.5 in both ATAC-seq and G4 CUT&Tag datasets were retained, ensuring sufficient signal in accessible chromatin. For the remaining peaks, M/A transformation was performed to assess relative enrichment. The average signal (M) was defined as ½ [log_2_(ATAC) + log_2_(G4Q)], and the difference in signal (A) as ½ [log_2_(G4Q) – log_2_(ATAC)]. Peaks were retained if M ≥ 1.0 and A ≥ 0.25, indicating substantial G4 signal above baseline accessibility. Finally, a stringent threshold of log_2_(G4Q) ≥ 3.0 was applied to identify high-confidence G4-enriched *loci*. This refinement procedure enriched for canonical G4 motifs by approximately 3.5-fold compared to the unfiltered peak set. To identify peaks with moderate signal strength in individual astrocyte cultures, assay-specific thresholds were applied to account for differences in signal distribution between ATAC-seq and G4 CUT&Tag. For ATAC-seq, peaks were considered moderately abundant if the mean normalized depth across all replicates for a given astrocyte culture was ≥ 5. For G4 CUT&Tag, due to lower coverage and discrete signal distribution, raw read depth was used, and peaks with a mean depth ≥ 2 were classified as above background. The presence or absence of each peak across astrocyte cultures was encoded as a binary matrix, which was exported in CSV format for downstream intersection and comparative analyses.

### Visualization and regression analysis.

Visualization of chromatin accessibility and G4 signal distributions was performed using multiple strategies. Annotation-category counts for ATAC-seq and G4 CUT&Tag peaks were displayed as stacked bar plots, with a secondary axis used to highlight G4 peak abundance. UpSet plots were generated to show the intersection of moderately abundant peaks across astrocyte cultures. Signal distributions and pairwise comparisons were visualized using violin, scatter, and segment plots created in Python (plotnine) and R (ggplot2). To model age-associated changes in chromatin accessibility and G4 signal, an ordinal regression framework was employed. Normalized coverage matrices were aggregated into a peak-by-sample matrix and log-transformed with an offset of 0.1. Using the VOOM approach, peak-wise means (μ_i_) and variances (v_i_) were calculated, followed by a locally weighted (lowess) fit to capture the mean–variance relationship. Individual age was encoded as an ordinal variable (e.g., 1 for age 22, 2 for age 24, etc.) and treated as a continuous predictor in the regression. The lmFit function from the limma package was used to estimate coefficients, with inverse variance weights (1/v_i_) applied. Empirical Bayes moderation was performed using the eBayes function. To account for sample-to-sample variability, each astrocyte culture was modeled as a random effect using the duplicate correlation function. In a separate analysis to detect sample-specific differences, all astrocyte samples were included, and a design matrix of culture-specific indicator variables was constructed. Differential comparisons between individual astrocyte cultures were performed using fitContrasts, and significance was assessed *via* moderated statistics from eBayes.

### Inter-modality comparisons.

To enable integrative comparisons between chromatin accessibility (ATAC-seq) and G4 enrichment (CUT&Tag), a conservative effect size estimation approach was employed using a custom shrinkage function. For each peak, the two-sided z-score associated with its differential p-value was used to derive an implied standard error. A lower (or upper) confidence bound on the log_2_ fold-change was then calculated at a user-defined confidence level (70% by default), and this “shrunk” estimate was used as the final effect size for all downstream analyses. Confidence intervals that included 0 were set to 0 for the “shrunken” estimate. Peaks were categorized into gain, loss, or neutral states based on whether the shrunken log_2_ fold-change exceeded assay-specific thresholds: ±0.25 for DDX5 knockdown contrasts and ± 0.05 for age-associated analyses. A quadrant-assignment function was used to combine ATAC-seq and G4 CUT&Tag states, labeling each peak with compound designations such as “DDX5 ATAC Gain | DDX5 G4 Gain” or “Age ATAC Loss | Age G4 Loss.” The number of peaks within each category was tabulated and incorporated into figure legends to aid in data interpretation.

### Gene set enrichment.

Gene set enrichment analysis was performed to interpret functional consequences of differential chromatin accessibility and G4 enrichment. Differential analysis results (topTables) for each comparison were merged with genomic peak annotations. Each peak was assigned a single “enrichment gene”: if the peak overlapped a genic region, the associated gene was retained; for intergenic peaks, the nearest upstream or downstream gene was selected based on proximity. The merged dataset was then filtered to include only peaks that passed prior quality and abundance thresholds, ensuring enrichment analyses focused on robust and biologically relevant *loci*. Over-representation analysis was conducted using the piano package. Differential p-values and log_2_ fold-changes for the enrichment genes were split by genomic context (genic vs. intergenic). Within each subset, hypergeometric tests were applied separately to upregulated (positive fold-change with significant p-value) and downregulated (negative fold-change with significant p-value) genes. Gene sets were restricted to sizes between 50 and 200 to reduce bias from extremely small or large categories. This produced four p-values per pathway: up-genic, down-genic, up-intergenic, and down-intergenic. To integrate evidence across contexts, the directional p-values were combined using Fisher’s method, yielding a unified over-representation p-value for upregulation and another for downregulation. In parallel, preranked enrichment analysis (GSEA) was conducted using the fgsea multilevel function. Genes were ranked by moderated t-statistics from differential models, and enrichment scores were computed separately for positive and negative rankings. Pathways containing 40–350 genes were included, and an epsilon of 1e–5 was used to stabilize estimates of extreme p-values. Empirical p-values were generated through adaptive permutations, and false discovery rates were calculated for all pathways. As with overrepresentation, GSEA was performed separately for genic and intergenic gene lists, and the four-resulting p-values per pathway were combined *via* Fisher’s method to generate a final significance score for both up- and down-regulated enrichment.

### DDX5 lentiviral transduction for RNA-sequencing.

For DDX5 overexpression, human astrocytes derived from a healthy 32-year-old patient were cultured in Poly-D-Lysine (PDL) coated T25 flasks at a seeding density of approximately 2 million cells per flask. Cells were transduced with a lentiviral vector expressing full-length human DDX5 (RefSeq: NM_001320595.2), obtained from VectorBuilder (Vector ID: VB221115–1626fqn), carrying either control mScarlet or mScarlet-tagged DDX5. Astrocytes were maintained in DMEM supplemented with 10% FBS, 1× penicillin-streptomycin, and defined astrocyte growth factors. Cells were infected at ~ 60–70% confluency, and the medium was replaced after 24 hours. mScarlet-DDX5 expression was confirmed by fluorescence microscopy 48–72 hours postinfection, and the following day, the cells were lysed in 1 mL of RLT buffer obtained from Qiagen, rapidly frozen, and stored at −80°C in preparation for RNA-sequencing.

### Sample preparation, library preparation, mRNA sequencing, and data analysis workflow.

RNA isolation was performed using the RNeasy Mini kit (Qiagen) following the manufacturer’s guidelines, with elution in a 30 μL volume. Subsequent library preparation was carried out, adhering to the standard QIAseq stranded mRNA Kit (Qiagen) protocol. Starting with 1 μg of initial material, mRNA was enriched and heat fragmented. Following the first and second strand synthesis, complementary DNA underwent end-repair and 3’ adenylation. Sequencing adapters were then ligated to the overhangs, and molecules carrying adapters were enriched through 11 cycles of PCR and purified using bead-based clean-up. Library quality control was performed using capillary electrophoresis (Tape D1000), and high-quality libraries were pooled based on equimolar concentrations. The concentration of the library pool was quantified using qPCR, and this optimal concentration was utilized to generate clusters on the surface of a flow cell.

Sequencing was conducted on a Nextseq instrument (Illumina Inc.) according to the manufacturer’s instructions, producing paired-end reads (2×75, 2×10).

Primary data analysis was carried out using CLC Genomics Server 22.0.2. Initial data processing involved trimming to remove potential read-through adapter sequences at the 3’ end, followed by quality score-based trimming and handling of reads containing ambiguous nucleotides (up to 2 per read). The trimmed reads were first mapped to the Human ribosomal RNA (rRNA) repetitive unit to assess the rRNA content in the samples. Subsequently, all reads were mapped to the Human genome GRCh38 with ENSEMBL GRCh38 version 98 annotation. For differential expression analysis, the ‘Empirical analysis of DGE’ algorithm within the CLC Genomics Workbench 22.0.2 was employed with default settings, utilizing the ‘Exact Test’ from the EdgeR Bioconductor package for two-group comparisons. Only genes with at least 10 counts summed across all samples were considered for unsupervised analysis. A variance stabilizing transformation was applied to the raw count matrix using the R package DESeq2 version 1.28.1. The principal component analysis included all genes, and variance values were calculated agnostically to pre-defined groups (blind = TRUE). Finally, hierarchical clustering was performed using the top 35 genes exhibiting the highest variance across samples.

### RNA-sequencing data analysis.

The RNA-sequencing data were subjected to analysis using iPathwayGuide, a tool provided by Adviata Bioinformatics. This analysis was performed within the context of pathways sourced from the KEGG (Kyoto Encyclopedia of Genes and Genomes) database, as well as gene ontologies obtained from the GO Consortium database. The analysis integrated these data sources to construct underlying topologies, which represent the network of genes and their directional interactions. These topologies were derived from the KEGG database utilizing iPathwayGuide, providing valuable insights into the biological pathways and processes influenced by the differential gene expression patterns identified through RNA sequencing.

### DDX5 knockdown in human astrocytes.

Human astrocytes cultured from a 24-year-old individual were transduced with a lentiviral shRNA vector targeting DDX5, obtained from VectorBuilder (Vector ID: VB900039–4212qgt). The construct expresses a U6-driven shRNA against DDX5 and an EGFP-T2A-Puromycin cassette under the PGK promoter. Cells were cultured in DMEM with 10% FBS, penicillin-streptomycin, and astrocyte growth factors, and infected at ~ 60–70% confluency. Media was replaced after 24 hours, and EGFP expression was confirmed by fluorescence microscopy at 48–72 hours. Cells were collected for downstream experiments, and knockdown efficiency was validated by ICC, JESS, and RT-qPCR.

### Immunocytochemistry.

Cultured human astrocytes were grown on Geltrex-coated chambered slides and subjected to immunofluorescence staining. After a preliminary wash with 1X Phosphate-Buffered Saline (PBS), the cells were fixed using 4% paraformaldehyde (PFA) for 10 minutes at room temperature, followed by PBS washes. Subsequent permeabilization and blocking were done using 5% normal goat serum, 1% Bovine Serum Albumin (BSA), and 0.3% Triton-X-100 for 45 minutes at room temperature. The astrocytes were then incubated with a primary antibody prepared in the blocking solution overnight at 4°C, washed thrice with PBS for 5 minutes each, and subsequently incubated with a fluorophore-tagged secondary antibody for 1 hour at room temperature. The slides were mounted with a DAPI-containing mounting medium (ProLong Diamond Antifade Mountant with DAPI, catalog No. P36962, Invitrogen, Thermo Scientific), enabling visualization using fluorescence microscopy.

### β-Galactosidase senescence staining.

β-Gal staining was performed with Senescence β-Galactosidase Staining Kit (Cell Signaling, #9860) according to the manufacturer’s instructions. Young and aged human astrocytes were seeded in 24-well plates. When the cells reach 70% confluency, the growth media was removed and cells were rinsed with 1X PBS and fixed with 1X fixative solution provided in the kit. After fixation, cells were washed twice with 1X PBS and were stained with freshly prepared β-Galactosidase staining solution at 37°C overnight. The β-Galactosidase positive cells were considered as senescent, and cells were counted from at least 4 randomly chosen fields from the cell plate.

### Capillary-based immunoassay via ProteinSimple^®^.

Protein expression of DDX5 was assessed using the JESS^™^ capillary-based immunoassay system (ProteinSimple, Bio-Techne; Catalog No. 004–650). Total protein lysates were prepared from human astrocytes using RIPA buffer (EMD Millipore, Catalog No. 20–188) supplemented with protease and phosphatase inhibitors (Cell Signaling Technology, Catalog No. 5872). Lysates were clarified by centrifugation at 12,000 rpm for 10 minutes at 4°C. Total protein concentration was estimated using a BCA assay, and samples were adjusted to a concentration of 1 μg/μL in ProteinSimple sample buffer. For each sample, a master mix was prepared by combining protein and master mix in a 4:1 ratio. The mix included 40 mM DTT and a fluorescent molecular weight standard. Samples were denatured at 95°C for 5 minutes and loaded into a 25-well separation module covering the 12–230 kDa range. DDX5 was detected using a rabbit monoclonal anti-DDX5 antibody (Cell Signaling Technology, Catalog No. 9877, clone D15E10) diluted in antibody diluent (ProteinSimple). An HRP-conjugated secondary anti-rabbit antibody was used for chemiluminescent detection. Signal intensities were quantified using Compass for SW software (v6.3.0, ProteinSimple), and results were expressed as peak area normalized to total protein as the loading control.

### Real-time qPCR.

Total RNA was extracted from young and aged primary human astrocytes using the RNeasy Mini kit (Qiagen; catalog no 74104). 1 μg of RNA was used to prepare the cDNA using iScript Reverse Transcription SuperMix (Bio-Rad; catalog no 1708840), according to the manufacturer’s protocol. Real-time qPCR was performed using a Bio-Rad CFX96 machine using SSoAdvanced Universal SYBR Green (Bio-Rad; catalog no 1725275) for visualization and quantification according to the manufacturer’s instructions. Primer sequences were: p16 (CDKN2A), forward: 5′–GGAGGCCGATCCAGGTCAT–3′, reverse: 5′–CACCAGCGTGTCCAGGAAG–3′; p21 (CDKN1A), forward: 5′–CAGCTGCCGAAGTCAGTTCC–3′, reverse: 5′–GTTCTGACATGGCGCCTCC–3′; p53 (TP53), forward: 5′–CGTGAGCGCTTCGAGATGTT–3′, reverse: 5′–TTGGACTTCAGGTGGCTGGA–3′; DEAD-box helicase 5 (DDX5), forward: 5′–ATTCTCCGCCGACCAAAACC–3′, reverse: 5′–CCGAAGCTGCACTACGGAAG–3′; DIRAS1, forward: 5′–GGGCCCAGGCTCGGT–3′, reverse: 5′–CCACCACGCGGTAATCGTT–3′; GAPDH, forward: 5′–CAGCCGCATCTTCTTTTGCG–3′, reverse: 5′–GCCCAATACGACCAAATCCGT–3′; Tubulin alpha 1a (TUBA1A), forward: 5′–GAAGCAGCAACCATGCGTGA–3′, reverse: 5′–TAGAGCTCCCAGCAGGCATT–3′. PCR conditions used were: 95°C for 3 minutes, followed by 40 cycles of 95°C for 10 seconds, and 60°C for 30 seconds. GAPDH and TUBA1A mRNA levels were used as an internal control for the target mRNAs. Normalization and relative fold change expression levels of all genes were calculated from the average threshold cycle number using the delta-delta Ct method.

### Fluorescence microscopy and image analyses.

Fixed cells were imaged using a Nikon A1R Confocal Laser Microscope, employing 20X, 40X, and 63X objectives. To ensure uniformity and comparability across all samples, imaging parameters, including light intensity, gain, and other relevant settings, were maintained constant throughout the imaging process. Subsequently, the acquired images were analyzed and quantified using ImageJ/Fiji software, enabling standardized measurements and evaluations of the experimental data.

### Statistical analysis.

The statistical analyses and tests were performed with ImageJ and GraphPad Prism software.

## Supplementary Material

Supplementary Files

This is a list of supplementary files associated with this preprint. Click to download.

- FigureS1.tif
- FigureS2.tif
- FigureS3.tif
- FigureS4.tif
- FigureS5.tif
- FigureS6.tif
- FigureS7.tif
- FigureS8.tif
- FigureS9.tif
- FigureS10.tif

## Data Availability

All sequencing datasets are deposited in the Genome Expression Omnibus under accession numbers GSE307272, GSE307273, and GSE307274. All other data supporting the findings of this study are available within the paper, in the supplementary information, or from the corresponding author upon request.
